# A protein structural study based on the centrality analysis of protein sequence feature networks

**DOI:** 10.1371/journal.pone.0248861

**Published:** 2021-03-29

**Authors:** Xiaogeng Wan, Xinying Tan

**Affiliations:** 1 College of Mathematics and Physics, Beijing University of Chemical Technology, Beijing, China; 2 The Fourth Center of PLA General Hospital, Beijing, China; Utrecht University, NETHERLANDS

## Abstract

In this paper, we use network approaches to analyze the relations between protein sequence features for the top hierarchical classes of CATH and SCOP. We use fundamental connectivity measures such as correlation (CR), normalized mutual information rate (nMIR), and transfer entropy (TE) to analyze the pairwise-relationships between the protein sequence features, and use centrality measures to analyze weighted networks constructed from the relationship matrices. In the centrality analysis, we find both commonalities and differences between the different protein 3D structural classes. Results show that all top hierarchical classes of CATH and SCOP present strong non-deterministic interactions for the composition and arrangement features of Cystine (C), Methionine (M), Tryptophan (W), and also for the arrangement features of Histidine (H). The different protein 3D structural classes present different preferences in terms of their centrality distributions and significant features.

## Introduction

Proteins are varied with their sequences, structures, and functions, the structures are encoded by their sequences, while the functions are decided by their structures [1–8]. Many studies have used protein sequence homology to predict the spatial structures of proteins [1]. Typical protein spatial structural prediction methods include artificial neural networks, nearest neighbor methods and support vector machines [1], e.g. the Chou-Fasman method [9], GOR (Garnier-Osguthorpe-Robson) [10], PHD [11], NNSSP [12], SymPsiPred [13] and CONCORD [14]. Other spatial structural prediction methods include homology modelling, threading, and ab initio methods [1]. Popular protein structural prediction servers are such as the SWISS-MODEL [15], RaptorX [16], ROBETTA [17], I-TASSER [18]. These methods predict the protein 3D structures providing their sequences. Recent studies also focus protein structural classification methods that can classify protein 3D structures into predefined classes [19–25]. Ding and Dubchak have used two new methods for protein fold classifications [19]. Edler and Grassmann have proposed a new protein fold classification method based on the feed forward neural networks (FFN) [20]. Huang et. al. have introduced three novel ideas for multiclass protein fold classification [21]. Jo et. al. have developed a deep learning network method (DN-Fold) to justify whether a given query-template protein pair belongs to the same structural fold [22]. Khan et. al. have used association rule mining technique–the ACO-AC to classify SCOP proteins into their correct folds [23]. Wei et al. have proposed a novel taxonomic method named PFPA for protein fold classification [24]. Wei and Zou have conducted a comprehensive review study surveying the recent computational methods, especially machine learning-based methods, in protein fold recognition [25].

Protein sequence feature extraction is a typical pre-process in protein classification studies [2–8]. These methods extract protein sequence features e.g amino acid composition and sequence arrangements [5], alignment scores [26], and physical properties of amino acids [27] into high dimensional real vectors or matrices, which are classified by spatial division methods such as the MSE (minimum-squared-error) hyperplanes [27, 28], convex hulls [28, 29], polygenetic trees [2–8] and Yau-Hausdorff distances [30, 31]. Typical protein sequence feature extraction methods are e.g. the natural vector (NV) [5], averaged property factors (APF) [27], protein map [3, 4], k-string dictionary [8], PseAAC [32], Pse-in-One [33], PSSM [26], etc.

Protein universe, include the intensive relations between its sequences, structures, and functions [2–8], together form a complex system, where the important behaviors of the system can be found by analyzing the pairwise-relations between its members [34, 35]. In research of complexity science, the systems are usually modelled as networks by abstracting the relations between their members [34, 35]. By modelling these systems into networks, we can further use network approaches [35] to analyze the behaviors of the systems. Bozhilova et. al. have performed a study on measuring the rank robustness in scored protein interaction networks [36]. Liu et. al. have performed a comprehensive review study on the various kinds of computational biological networks [37], where they summarize the various biological networks and network-based approaches from recent studies and with guidelines to diverse biological applications.

In this paper, we model the protein universe using complex networks, where we believed there exist abundant information behind the relations between the various protein sequence features. We use classic centrality measures to analyze weighted networks constructed from the pairwise-relations between the sequence features, and use Welch T-tests to identify the significant features for the different types of protein 3D structures, where we find both similarities and differences between the different types of structures. This study approaches the protein structural analysis from a new complex network prospect, which makes up the deficiency of tradition protein classifiers that they focus on high-dimensional divisions of feature points but neglect the relations between these features. The methods and results of this study are useful for future development of new protein structural predictors or classifiers by considering the significant features for the different structures, or the exploration of significant features for deeper protein structural levels. The results may help us gain more understanding on the influences between protein sequences and structures.

The paper is organized as follows. In the Materials and methods section, we introduce the protein sequence feature extraction methods, connectivity and centrality measures used in our study. In the Results section, we use protein sequence data from CATH and SCOP database to demonstrate the centrality analysis. The similarities and differences between the different structures and interpretations of the connectivity measures are discussed in the Discussion section, and the conclusions are drawn in the Conclusions section.

## Materials and methods

In this section, we introduce the protein sequence feature extraction methods, connectivity and centrality measures as well as the Welch T-test used in this study.

### Protein feature extraction methods

#### Natural vector (NV)

Natural vector (NV), introduced by Yau [5], is a 60-dimensional real vector that uniquely characterizes the composition and sequence arrangement of a protein sequence by [5]:

$$\langle n_{A},n_{R},\ldots ,n_{V},\mu_{A},\mu_{R},\ldots ,\mu_{V},D_{2}^{A},D_{2}^{R},\ldots ,D_{2}^{V}\rangle .$$ (1)

where *n*_*k*_ (N feature) is the number of the amino acid k in the protein sequence, $\mu_{k}=\frac{T_{k}}{n_{k}}$ (*μ* feature) is the arithmetic mean value for the total distances of the *k*-type amino acids from the origin, where $T_{k}=\sum_{i=1}^{n_{k}}s[k][i]$ is the total distance of every amino acid *k* to the origin and *s*[*k*][*i*] is the distance from the first amino acid (regarded as origin) to the *i*-th amino acid *k* in the sequence; $D_{2}^{k}$ (D feature) is the 2^nd^ order normalized central moment defined by: $D_{j}^{k}=\sum_{i=1}^{n_{k}}\frac{{\left(s[k][i]-\mu_{k}\right)}^{j}}{n_{k}^{j-1}n^{j-1}}$ [5], *j* = 1,2,…,*n*_*k*_, *k* = A, R, N, D, C, Q, E, G, H, I, L, K, M, F, P, S, T, W, Y, V represent the 20 types of amino acids (the names, classifications of the 20 types of amino acids are shown in S1 Table).

#### Averaged property factor (APF)

Averaged property factor (APF), introduced by Rackovsky [27], is a 10-diemsional vector extracts the 10 important physical properties of amino acids in a protein sequence [27]:

$$V_{S}=({\langle f^{\left(1\right)}\rangle }_{S},{\langle f^{\left(2\right)}\rangle }_{S},\ldots ,{\langle f^{\left(10\right)}\rangle }_{S})$$ (2)

where S denotes the protein sequence and ${\langle f^{\left(m\right)}\rangle }_{S}=\frac{1}{N_{S}}\sum_{n=1}^{N_{S}}f_{n}^{\left(m\right)}$ is the sequence-average of the *m*-th property factor, *N*_*S*_ is the number of residues in *S*, $f_{n}^{\left(m\right)}$ is the value for the *m*-th property of amino acid *n*, *m* = 1,2,…,10 correspond to the 10 physical properties [27, 38–44]. Details of the 10 physical properties are shown in S2 Table, the values of these properties can be found in Table V of [38].

#### Pseudo amino acid composition (PseAAC)

Pseudo amino acid composition (PseAAC), introduced by Chou [32, 45], is a 20 + *λ* (integer *λ*≥0) dimensional real-vector represent the composition and the sequence arrangements of the 20 types of amino acids in a protein sequence [32, 45–49]:

$$X={\left[x_{1},\cdots ,x_{20},x_{20+1},\cdots ,x_{20+\lambda }\right]}^{T},$$ (3)

where

$$x_{u}=\left\{ \begin{array}{cc} \frac{f_{u}}{\sum_{i=1}^{20}f_{i}+\omega \sum_{j=1}^{\lambda }\theta_{j}}, & \left(1\leq u\leq 20\right)\\ \frac{\omega \theta_{u-20}}{\sum_{i=1}^{20}f_{i}+\omega \sum_{j=1}^{\lambda }\theta_{j}}, & \left(20+1\leq u\leq 20+\lambda \right) \end{array}\right.$$ (4)

*f*_*u*_ is the normalized occurrence frequency for the 20 amino acids in the protein [45], *θ*_*j*_ is the j-tier sequence correlation factor (computed by Eqs (2–4) in S1 Text) of the protein sequence, *λ* is a non-negative integer no greater than the length of the protein sequence, *w* is the weight factor for the sequence order effect e.g. *w* = 0.05 [45] as used in our analysis, other *w* values are plausible upon user preferences.

When *λ* = 0, PseAAC is the original occurrence frequency for the 20 types of amino acids; when *λ*>0, the first 20 components *x*_*u*_(1≤*u*≤20) are the composition effects modified by the weighted terms for the sum of the *λ*-tier correlation term $\sum_{j=1}^{\lambda }\theta_{j}$, the additional *λ* components reflect the sequence arrangement effects. The optimum choice of *λ* can be tested by the Covariant Discriminant Algorithm (CDA) [45]. In our analysis, we test the CATH and SCOP data with 0<*λ*≤20 (since there are 20 types of amino acids, we consider protein sequences no shorter than 20 amino acids residues), we find *λ* = 10 is optimal for SCOP, while the CATH data admits no great differences in the CDA tests when *λ* varies from 1 to 20. Studies show that the slight inaccuracies aroused by using a same optimal *λ* for different datasets are trivial [45]. Hence, we consider *λ* = 0 and *λ* = 10 for both CATH and SCOP in our analysis. Details of the PseAAC features are shown in S1 Text.

### Data download and sequence feature extraction

Since high similarity protein sequences may get similar or repetitive feature elements, which are redundant in the relationship analysis of feature series, therefore we use the lowest 30% similarity protein sequences (can be filtered in Protein Data Bank) with CATH and SCOP classifications to perform the analysis. The 30% similarity is low enough to avoid the redundancy, while ensuring sufficient data to achieve good statistics. Here, we focus on the top structural categories of CATH and SCOP rather than other deeper levels because of two main reasons. First, because the data covers the entire database that is in great amount and the feature vectors are in high dimensions, it requires intensive computation for the relationship and centrality analyses for the high dimensional large data. Secondly, the top structural categories are the basic classifications for protein structures, explorations of deeper structural levels should be performed on the ground of the basic categories, i.e. we need to first get the knowledge of the top categories and then analyze the deeper levels. Results on the top categories will be the solid foundations for future deeper level analysis.

In our study, we use the NV, APF and PseAAC to extract the protein sequence features and use connectivity measures to analyze the relations between these features. Since different features may get different value ranges, thus different magnitudes of the relationships, therefore we consider features of the six types, namely the N, *μ*, D features of NV, the APF features, and the PseAAC with *λ* = 0 and *λ* = 10, we separately perform the relationship analysis for the six types of features.

### Random permutation on feature series

For a set of N protein sequences, the *K* dimensional feature vectors together form a *N*×*K* feature matrix, where *K* = 20 for N, *μ*, D and PseAAC (*λ* = 0), *K* = 10 for APF, and K = 30 for PseAAC (*λ* = 10). The rows of the feature matrix are the feature vectors of K dimensions, while the columns are feature series *X*_1_,*X*_2_,…,*X*_*K*_ for the K feature factors. For an instance, the j-th column is the feature series *X*_*j*_ formed by elements from the j-th feature factor, *j* = 1,2,…,*K*. The feature series are real-valued series presenting the states of specific feature factors. We treat these feature series as real-world time series and use connectivity measures to analyze pairwise-relations between these features. For the set of N protein sequences, all feature series have the same length N, the *i*-th position of the feature series are the feature elements of the *i*-th protein, *i* = 1,2,…,*N*. Since the protein orders are embodied by the arrangements of the rows, and different protein orders may affect the values of the relationships, therefore to eliminate this protein order effect, we randomly permute the rows of the feature matrix in order to rearrange the orders of the proteins, the relationship and centrality analysis are performed on every random permutation of the feature matrices. We use the average standard deviations over the random permutations to test the robustness of the results. Since the purpose of random permutations is to eliminate the protein order effects, therefore, larger permutation number will get better results. However, the permutation number should balance with the relationship and centrality computations. We have tested with a series of permutation numbers from 10, 20 to 100, where the average standard deviation results are shown in S3 Table, from which results we can see that the variations of the standard deviations are small, which prove the robustness of our results. Here, we use 100 random permutations to perform the analysis which is large enough for our analysis.

### Relationship analysis among feature series

In this section, we recall the connectivity measures that are used to analyze the relations between protein sequence features.

#### Absolute correlation (CR)

For a structural class of *N*_*s*_ proteins, we get an *N*_*s*_×*K* dimensional feature matrix, K is the feature dimension, *s* denotes the structural classes, *s* = 1,2,3 for the mainly *α*, mainly *β*, and the mixed *α* and *β* classes of CATH or *s* = *a*, *b*, *a*/*b*, *a*+*b* for the all *α*, all *β*, *α*/*β*, *α*+*β* classes of SCOP. The rows are feature vectors, while the columns are feature series of length *N*_*s*_. The *j*-th feature series (column) are denoted as

$$X_{j}=\left\{x_{1,j},x_{2,j},\ldots ,x_{N_{s},j}\right\},j=1,2,\ldots ,K.$$ (5)

where *x*_*i*,*j*_ is the *i*-th element of the *j*-th feature series (i = 1,2,…, *N*_*s*_, j = 1,2,…,K). The K feature series are then denoted as {*X*_1_,*X*_2_,…,*X*_*K*_}.

For each structural class, we get a K×K dimensional absolute correlation matrix:

$$R^{\prime }=\left(\begin{array}{cccc} r_{11}^{\prime } & r_{12}^{\prime } & \cdots & r_{1,K}^{\prime }\\ r_{21}^{\prime } & r_{22}^{\prime } & \cdots & r_{2,K}^{\prime }\\ \vdots & \vdots & \ddots & \vdots \\ r_{K,1}^{\prime } & r_{K,2}^{\prime } & \cdots & r_{K,K}^{\prime } \end{array}\right)$$ (6)

where $r_{ij}^{\prime }=|r_{ij}|,$ and $r_{ij}=\frac{Cov(X_{i},X_{j})}{\sqrt{Var(X_{i})Var(X_{j})}}=\frac{E[(X_{i}-EX_{i})(X_{j}-EX_{j})]}{\sqrt{Var(X_{i})Var(X_{j})}}$ is the correlation between *X*_*i*_ and *X*_*j*_ (i,j = 1,2,…,K). This matrix is symmetric i.e. R = *R*^*T*^ (T denotes matrix transpose), it depicts the symmetric linear relations between feature series. The values of the absolute correlations are ranged between 0 and 1, which reflect the strength of the linear relations, where higher values indicate the stronger the linear relations.

#### The normalized mutual information rate (nMIR)

Similar to CR, we can get a K×K nMIR matrix for each of the structural classes:

$$I^{\prime }=\left(\begin{array}{cccc} I_{11}^{\prime } & I_{12}^{\prime } & \cdots & I_{1,K}^{\prime }\\ I_{21}^{\prime } & I_{12}^{\prime } & \cdots & I_{2,K}^{\prime }\\ \vdots & \vdots & \ddots & \vdots \\ I_{K,1}^{\prime } & I_{K,2}^{\prime } & \cdots & I_{K,K}^{\prime } \end{array}\right),$$ (7)

where $I_{ij}^{\prime }=\left\{ \begin{array}{c} I(X_{i},X_{j})/H_{max},\;i\neq j,\\ H(X_{i})/H_{max},\;i=j, \end{array}\right.$ is the nMIR value between *X*_*i*_ and *X*_*j*_ (*i*,*j* = 1,2,…,*K*) [36], $H_{max}=\underset{i}{\max}\;H(X_{i})$ is the maximum entropy for all *X*_*i*_ (i = 1,2,…,K), $I_{ij}=\left\{ \begin{array}{c} I(X_{i};X_{j}),\;i\neq j\\ H(X_{i}),\;i=j \end{array}\right.$ is the mutual information rate between *X*_*i*_ and *X*_*j*_, and

$$I\left(X_{i};X_{j}\right)=\sum_{\alpha ,\beta }p\left(x_{i}=\alpha ,x_{j}=\beta \right)\log\frac{p\left(x_{i}=\alpha ,x_{j}=\beta \right)}{p\left(x_{i}=\alpha \right)p\left(x_{j}=\beta \right)},\;i\neq j,i,j=1,2,\ldots ,K,$$ (8)

when *i* = *j* it degenerates to the Shannon Entropy of *X*_*i*_ [50]:

$$H(X_{i})=\sum_{\alpha }p(x_{i}=\alpha )\log\frac{1}{p(x_{i}=\alpha )}=-\sum_{\alpha }p(x_{i}=\alpha )\log\;p(x_{i}=\alpha ).$$ (9)

The matrix I′ is symmetric in that I(*X*_*i*_; *X*_*j*_) = I(*X*_*j*_; *X*_*i*_), *i*,*j* = 1,2,…,*K*. The nMIR values, ranged between 0 and 1, evaluate the normalized uncertainties eliminated for *X*_*i*_ when knowing *X*_*j*_, i.e. the “common information” shared by the two series, whereas the Shannon entropy of *X*_*i*_ indicates the uncertainties of *X*_*i*_ itself. The nMIR is a model-free measure that evaluates mutual relations no matter linear or not. Higher nMIR values may indicate stronger symmetric relations between the feature series [50].

#### Transfer Entropy (TE)

TE is a fundamental information transfer measure that evaluates the asymmetric interaction between feature series [51]. It is a bivariate measure defined by [51]:

$${TE}_{X_{j}\to X_{i}}=\sum_{\alpha ,\beta ,\gamma }p(X_{n+1,i}=\gamma ,X_{n,i}^{\left(k\right)}=\alpha ,X_{n,j}^{\left(l\right)}=\beta )log\frac{p(X_{n+1,i}=\gamma |X_{n,i}^{\left(k\right)}=\alpha ,X_{n,j}^{\left(l\right)}=\beta )}{p(X_{n+1,i}=\gamma |X_{n,i}^{\left(k\right)}=\alpha )},$$ (10)

where *X*_*i*_, *X*_*j*_ are feature series (*i*,*j* = 1,2,…,*K*), *X*_*n*+1,*i*_ denotes the state of *X*_*i*_ at time *n*+1 (the *n*+1-th element of feature series *X*_*i*_), *γ* is the state value of *X*_*n*+1,*i*_, $X_{n,i}^{\left(k\right)}=(X_{n,i},X_{n-1,i},\ldots ,X_{n-k+1,i})$ and $X_{n,j}^{\left(l\right)}=(X_{n,j},X_{n-1,j},\ldots ,X_{n-l+1,j})$ are embedding vectors for the lagged variables of *X*_*i*_, *X*_*j*_, **α** and **β** are states of $X_{n,i}^{\left(k\right)}$ and $X_{n,i}^{\left(l\right)}$, *l*,*k* usually take values with *l* = *k* (basic requirement for information transfer detection) are the maximum time lags for $X_{n,i}^{\left(k\right)}$, $X_{n,j}^{\left(l\right)}$ [51]. The summation in (10) runs over all possible combinations of the states of *X*_n+1_, $X_{n}^{\left(k\right)}$ and $Y_{n}^{\left(l\right)}$. TE is asymmetric, where TE_Y→X_ indicates the dependence of X on Y [51]. In practice, the values of *l*,*k* influence not only the quality of information transfer detection, but also the computation speed. Larger *l*,*k* may detect deeper levels of information transfers, but have longer computation times. Here, we use the most computational efficient nearest neighbor estimator to estimate TE [53]. Although there is no fixed rules for the choices of *l*,*k* and they often depend on the data types to be analyzed, however, a rule of thumb is to use small *l*,*k* for discontinuous observations, but large *l*,*k* for smoothly changing flows [52, 53]. In practice, *l* = *k* = 5 is recommended for real world data analysis. Larger *l*,*k* are feasible, but may result in more intensive computation of TE, which is often impractical and time consuming. We take the PseAAC features (*λ* = 10) as an example to illustrate the influences of *l*,*k* with changing values in {1,5,10,15,20}. The resulting 30×30 relationship matrices are plotted into heat-maps as shown in S1 Fig. In S1 Fig, the heat-maps present the magnitudes of TE, where the different choices of *l*,*k* present similar relationship results. Since the feature series are real-valued discontinuous observations rather than smoothly changing flows, *l* = *k* = 5 is enough for our analysis. Larger parameters may get similar results but longer computation time.

#### Time-shifted surrogatesmutual information rate (nMIR)

Information transfer measures often contain bias in the information transfer detection [53–56], thus bias-correction is necessary to amend the TE values. The bias-correction is to make a significance threshold, where information transfers surpass this threshold are deemed valid. In practice, the bias is often deducted from the information transfer value by using the threshold, the remain value is used as the corrected information transfer value. Time-shifted surrogate is a popular technique for bias-correction [53, 56]. Let *X*_*i*_, *X*_*j*_ be two feature series, we shuffle the index of *X*_*i*_ while keeping *X*_*j*_ unchanged in order to obtain a surrogate of *X*_*i*_ [53–56]. Then, apply TE on *X*_*j*_ and the surrogate of *X*_*i*_, we get ${TE}_{X_{j}\to X_{i}}(q)$, q is the surrogates’ index. The bias-corrected TE is given by [54, 55]

$${TE}_{C,X_{j}\to X_{i}}={TE}_{X_{j}\to X_{i}}-\underset{q}{\max}\left\{{TE}_{X_{j}\to X_{i}}(q)\right\}.$$ (11)

We use typical parameter q = 10 [54, 55] for all TE computations. The threshold $\underset{q}{\max}\left\{{TE}_{X_{j}\to X_{i}}(q)\right\}$ is varied between series to series. The principle of this threshold is to filter TE, specific threshold values are ineffective, but the information transfers surpass this threshold matter. In practice, we set ${TE}_{C,X_{j}\to X_{i}}=0$ when ${TE}_{C,X_{j}\to X_{i}}<0$, this means that there is no significant information transfer from *X*_*j*_ to *X*_*i*_. The final *K*×*K* bias-corrected TE matrix is given by

$$TE_{C}=(\begin{array}{cccc} TE_{C,1\to 1} & TE_{C,1\to 2} & \cdots & TE_{C,1\to K}\\ TE_{C,2\to 1} & TE_{C,2\to 2} & \cdots & TE_{C,2\to K}\\ \vdots & \vdots & \ddots & \vdots \\ TE_{C,K\to 1} & TE_{C,K\to 2} & \cdots & TE_{C,K\to K} \end{array})$$ (12)

where *K* is the feature dimension, and *TE*_*C*,*j*→*i*_ is the bias-corrected TE for *X*_*j*_→*X*_*i*_, *i*,*j* = 1,2,…,*K*.

#### Independence of the three measures

We use CR, nMIR and TE to analyze the pairwise-relations between the features. The three measures are mutually independent with each other. CR and nMIR measure the symmetric relations between feature series, while TE evaluates the asymmetry information transfers between the series. Both CR and nMIR are scaled between 0 and 1, where CR indicates symmetric linear dependence between feature series, while nMIR is a model-free measure that evaluates symmetric relations no matter linear or not. TE is a directed information transfer measure whose value is independent with CR and nMIR. A positive TE value indicates the existence of directed influences from one series to another, where the dependence is usually non-deterministic. TE will be vanished for deterministic relations. In fact, a high symmetric value may not correspond with a high asymmetric value, and vice versa. Detailed discussions of these measures are shown in the Discussion section.

### Network construction and centrality analysis

In this study, we use the relationship matrices for different features to construct weighted networks, where we use CR and nMIR matrices to construct undirected networks, and use TE matrices to construct directed networks. For a network of *K* nodes, the nodes are the protein sequence features, while the links are the relations between these features. Since there are 100 random permutations of feature series, we will get 100 matrices for each kind of relations. For an example of the *K*×*K* dimensional CR matrix *R*′ (K is the number of features), we set *A* = *R*′ as the adjacency matrix, a link is drawn between the node *i* (the *i*-th feature) and *j* (the *j*-th feature) with weight $r_{ij}^{\prime }$ if $a(i,j)=r_{ij}^{\prime }>0$, otherwise no link is drawn between nodes *i* and *j* (*i*≠*j*,*i*,*j* = 1,2,…,*K*). The networks of nMIR relations are similarly constructed. Since CR and nMIR matrices are symmetric, the CR and nMIR networks are all undirected. However, the TE networks are directed, a link is drawn from node *j* (the *j*-th feature) to node *i* (the *i*-th feature) with weight *TE*_*C*,*j*→*i*_ if *a*(*i*,*j*) = *TE*_*C*,*j*→*i*_>0; otherwise, there is no link from node *j* to node *i* (*i*≠*j*,*i*,*j* = 1,2,…,*K*). We use this method to construct weighted networks for all top hierarchical classes of CATH and SCOP and for all types of features. In the networks of N, *μ*, D and PseAAC (*λ* = 0) features, there are 20 nodes correspond to the features of the 20 amino acids, while the networks of APF and PseAAC (*λ* = 10) features separately contain 10 and 30 nodes, correspond to the 10 physical properties and the 30 dimensional PseAAC features (1–20 dimensions: proportional compositions of the 20 amino acids, 21–30 dimensions: the 10-tier correlations for the sequence order effects).

We use classic centrality measures with weighted adjacency matrices to analyze the importance of the features (nodes). For a network of K nodes and weighted adjacency matrix *A* = (*a*_*ij*_)_*K*×*K*_, the centrality vector is represented by *y* = (*y*_1_.⋯,*y*_*k*_)^*T*^, where *y*_*j*_ is the centrality of the node j, *j* = 1,2,…,*K*. Since the centralities evaluate the importance of the nodes, specific values of centrality are ineffective, but the comparisons over all magnitudes matter [35]. Nodes with higher centralities than others are deemed as more important. Here, we consider both undirected and directed networks, where we use degree and eigenvector centralities for undirected networks, and use in and out degree centrality, Katz centrality and PageRank for directed networks.

#### Centrality measures for undirected networks

*Degree centrality*. For undirected networks, the adjacency matrices are symmetric. The degree centrality of weighted networks is defined by the sum of the weights for the links connecting to the node. Let *A* = (*a*_*ij*_)_*K*×*K*_ be the weighted adjacency matrix for an undirected network, the degree centrality is given by

$$y_{j}=\sum_{i=1}^{K}a_{ij}=\sum_{i=1}^{K}a_{ji},\;j=1,2,\ldots ,K.$$ (13)

where *a*_*ij*_ is the weight of the link connects nodes *i* and *j*. Since A is symmetric, *a*_*ij*_ = *a*_*ji*_, the degree centrality of a node j equals both the sum of the j-th column and the sum of the j-th row of A [35].

*Eigenvector centrality*. Degree centrality is the simplest centrality measure, which does not consider the influences of the neighbors. The eigenvector centrality of a node j is defined as the sum of the eigenvector centralities of its neighbors [35]:

$$y_{j}=k_{1}^{-1}\sum_{i=1}^{K}a_{ji}y_{i}=k_{1}^{-1}\sum_{i=1}^{K}a_{ij}y_{i},\;j=1,2,\ldots ,K.$$ (14)

In matrix notation, the eigenvector centrality ***y*** satisfies *A****y*** = *k*_1_***y***, where *k*_1_ is the leading eigen value of the weighted adjacency matrix A, ***y*** is the right leading eigenvector of A [35]. Theoretically, eigenvector centrality can be used for both undirected and directed networks, but in practice, it is easier to apply for undirected networks [35], because, in directed networks, the adjacency matrix is asymmetric, which have both left and right eigenvectors that result in two leading eigenvectors for each directed network. Although right eigenvectors are more appropriate to be used as centralities [35], but we still need to justify which type of eigenvectors should be used when dealing with directed networks. Moreover, in directed networks, there are also problems for the nodes without in-going links, which may get inappropriate zero centralities no matter how many out-going links it has [35]. Therefore, the eigenvector centrality is usually used for undirected networks, and we use it for only undirected network in our analysis.

#### Centrality measures for directed networks

*In and out degree centralities*. In directed networks, the links are directed and the adjacency matrices are asymmetric. The direction of the adjacency matrix is indicated from the columns to the rows, e.g. the adjacency element *a*_*ij*_ is the weight for the link from node *j* to node *i*. In weighted networks, the in-degree centrality is defined as the sum of the weights for all in-going links point to the node, which is represented by

$$y_{j}^{in}=\sum_{i=1}^{K}a_{ji},\;j=1,2,\ldots ,K.$$ (15)

where *a*_*ji*_ is the weight of the link from node *i* to node *j*. Similarly, the out-degree is defined as the sum of the weights for all out-going links from this node to the other nodes:

$$y_{j}^{out}=\sum_{i=1}^{K}a_{ij},\;j=1,2,\ldots ,K.$$ (16)

*Katz centrality*. In and out degree centralities are the simplest centrality measures for directed networks, which do not account the neighbor effects. Similar to the eigenvector centrality, Katz centrality considers the centralities of the neighbors. Katz centrality is an improvement of the eigenvector centrality when applying for directed networks, which is defined by [35].

$$y={\left(I-\alpha A\right)}^{-1}\beta ,$$ (17)

I is the *K*×*K* dimensional identity matrix, *α* is a real positive value empirically slightly smaller than the leading eigenvalue of the weighted adjacency matrix A, ***β*** is a *K*-dimensional vector as the “free” centrality given in the iterative process when solving the problems of eigenvector centrality in directed networks [35]. If we use ***β*** = **1** (a *K*-dimensional 1-vector), the expression of Katz centrality is reduced to [35].

$$y={\left(I-\alpha A\right)}^{-1}1.$$ (18)

*PageRank*. Katz centrality also has drawbacks. If a node of very high centrality points to a great number of neighbors, the out-neighbors of the high centrality node will inherit improper high centralities by Katz [35]. This issue is solved by the PageRank. PageRank is a centrality measure for directed networks, which can be expressed by [35]:

$$y={D(D-\alpha A)}^{-1}1,$$ (19)

where *D* = (*d*_*ii*_)_*K*×*K*_ is a diagonal matrix with diagonal element $d_{ii}=\underset{}{\max}\left(1,k_{i}^{out}\right)$, here $k_{i}^{out}$ is the out-degree (sum of the weights for all out-going links) of the i-th node.

#### Normalization of centrality values

We compute the centrality values for the weighted networks of all features and all top hierarchical class of CATH and SCOP. To make fair comparison of the centralities, the original centrality values are normalized by dividing the maximum centrality value in the same networks. By this normalization, all centrality values are scaled between 0 and 1, where the maximum centrality value is normalized to 1. The higher the normalized centralities approximate 1, the more important the nodes (features).

### Standard deviation analysis

The centralities are computed for every random permutation of feature series. To evaluate the robustness of the results, we compute the average standard deviations for the normalized centrality results over all random permutations. Take the degree centrality in the CR network of N features (CATH) as an example. The CATH data has three top hierarchical classes, which correspond to three CR networks. For each structural class, the networks contain 20 nodes for the N features of the 20 amino acids. For every random permutation, we get a 20-dimensional centrality vector for each structural class. Therefore, we get 100 such vectors for the 100 random permutations. The standard deviation is computed for the normalized centrality over the 100 permutations, which results in a 20-dimensional vector *v*_*σ*_ = (*σ*_1_,*σ*_2_,⋯,*σ*_20_) for the standard deviations, *σ*_*i*_ is the standard deviation of the node *i*. We compute the average of the vectors *v*_*σ*_ for all structural classes, which result in $\bar{\sigma_{R,D}}$ as the final mean standard deviation value for the degree centrality of the CR networks. The mean standard deviations are computed for all types of networks and centralities. The results are shown in Tables 1–4.

**Table 1:** The mean standard deviation results for the centralities of undirected networks (CATH).

Centrality measures	Mean standard deviations in undirected CR and nMIR networks (by features)					
	N	*μ*	D	APF	PseAAC (*λ* = 0)	PseAAC (*λ* = 10)
**Degree centrality** ($\bar{\sigma_{R,D}}$)	1.08×10^−15^	1.26×10^−15^	1.28×10^−15^	9.45×10^−16^	7.52×10^−16^	7.09×10^−16^
**Eigenvector centrality** ($\bar{\sigma_{R,E}}$)	1.20×10^−15^	1.26×10^−15^	1.34×10^−15^	1.01×10^−15^	8.47×10^−16^	6.98×10^−16^
**Degree centrality** ($\bar{\sigma_{I,D}}$)	7.71×10^−16^	9.89×10^−16^	8.62×10^−16^	8.22×10^−16^	7.70×10^−16^	8.25×10^−16^
**Eigenvector centrality** ($\bar{\sigma_{I,E}}$)	8.56×10^−16^	1.00×10^−15^	8.57×10^−16^	8.07×10^−16^	7.50×10^−16^	8.20×10^−16^

This table shows the mean standard deviation results for the normalized degree and eigenvector centralities of the undirected CR and nMIR networks. The $\bar{\sigma_{R,D}}$ and $\bar{\sigma_{R,E}}$ denote the mean standard deviations for the degree and eigenvector centralities in CR networks, while $\bar{\sigma_{I,D}}$ and $\bar{\sigma_{I,E}}$ denote the mean standard deviations for the degree and eigenvector centralities in nMIR networks.

**Table 2:** The mean standard deviation results for the centralities of directed networks (CATH).

Centrality measures	Mean standard deviations in directed TE networks (by features)					
	N	*μ*	D	APF	PseAAC (*λ* = 0)	PseAAC (*λ* = 10)
**In degree centrality** ($\bar{\sigma_{T,DIN}}$)	1.09×10^−1^	2.06×10^−1^	2.07×10^−1^	2.87×10^−1^	2.14×10^−1^	5.88×10^−2^
**Out degree centrality** ($\bar{\sigma_{T,DOUT}}$)	1.07×10^−1^	2.03×10^−1^	2.08×10^−1^	2.90×10^−1^	2.14×10^−1^	6.65×10^−2^
**Katz centrality** ($\bar{\sigma_{T,Katz}}$)	9.61×10^−2^	2.10×10^−1^	2.01×10^−1^	2.73×10^−1^	1.99×10^−1^	6.63×10^−2^
**PageRank centrality** ($\bar{\sigma_{T,PR}}$)	8.99×10^−2^	1.93×10^−1^	1.93×10^−1^	2.47×10^−1^	1.79×10^−1^	6.01×10^−2^

This table shows the mean standard deviation results for the normalized in ($\bar{\sigma_{T,DIN}}$) and out ($\bar{\sigma_{T,DOUT}}$) degree centralities, Katz ($\bar{\sigma_{T,Katz}}$) and PageRank ($\bar{\sigma_{T,PR}}$) centralities of the directed TE networks.

**Table 3:** The mean standard deviation results for the centralities of undirected networks (SCOP).

Centrality measures	Mean standard deviations in undirected CR and nMIR networks (by features)					
	N	*μ*	D	APF	PseAAC (*λ* = 0)	PseAAC (*λ* = 10)
**Degree centrality** ($\bar{\sigma_{R,D}}$)	9.85×10^−16^	1.10×10^−15^	1.13×10^−15^	7.94×10^−16^	7.20×10^−16^	7.24×10^−16^
**Eigenvector centrality** ($\bar{\sigma_{R,E}}$)	1.08×10^−15^	1.22×10^−15^	1.19×10^−15^	9.19×10^−16^	8.01×10^−16^	7.15×10^−16^
**Degree centrality** ($\bar{\sigma_{I,D}}$)	7.04×10^−16^	9.51×10^−16^	7.99×10^−16^	9.32×10^−16^	7.60×10^−16^	7.74×10^−16^
**Eigenvector centrality** ($\bar{\sigma_{I,E}}$)	9.21×10^−16^	9.64×10^−16^	8.03×10^−16^	1.03×10^−15^	8.19×10^−16^	7.87×10^−16^

This table shows the mean standard deviation results for the normalized degree and eigenvector centralities of the undirected CR and nMIR networks. The notations are similarly defined as in Table 1.

**Table 4:** The mean standard deviation results for the centralities of directed networks (SCOP).

Centrality measures	Mean standard deviations in directed TE networks (by features)					
	N	*μ*	D	APF	PseAAC (*λ* = 0)	PseAAC (*λ* = 10)
**In degree centrality** ($\bar{\sigma_{T,DIN}}$)	1.33×10^−1^	1.80×10^−1^	1.92×10^−1^	2.84×10^−1^	2.07×10^−1^	5.96×10^−2^
**Out degree centrality** ($\bar{\sigma_{T,DOUT}}$)	1.33×10^−1^	1.87×10^−1^	1.89×10^−1^	2.83×10^−1^	2.00×10^−1^	6.74×10^−2^
**Katz centrality** ($\bar{\sigma_{T,Katz}}$)	1.19×10^−1^	1.77×10^−1^	1.77×10^−1^	2.72×10^−1^	1.86×10^−1^	6.72×10^−2^
**PageRank centrality** ($\bar{\sigma_{T,PR}}$)	1.34×10^−1^	1.64×10^−1^	1.63 ×10^−1^	2.45×10^−1^	1.70×10^−1^	6.12×10^−2^

This table shows the mean standard deviations for the normalized in and out degree centrality, Katz and PageRank centralities of the directed TE networks. The notations are similarly defined in Table 2.

### Significance of centralities

Centralities depict the importance of the nodes in networks. To identify the significant centralities among the features, we perform pairwise Welch T-tests between these features. Since the relationship and centrality analysis are performed for the 100 random permutation of feature series, we get 100 centrality results for each node in the different networks. For a network of K features, *Y*_*i*_ denotes the centrality of feature *i*, the 100 centrality values of feature *i* can be viewed as 100 samples for *Y*_*i*_. Since the sample size *n*_*i*_ = 100 is large, these samples can be viewed as follow normal distribution $N(\mu_{i},\sigma_{i}^{2})$, where *μ*_*i*_ and $\sigma_{i}^{2}$ are the expectation and standard deviations of centrality *Y*_*i*_. To test the significant differences between these features, we first use the Levene-test to check the homogeneity of the variance for the different centralities. For the network of K features (nodes), the Levene-test uses F-statistics [52]:

$$F=\frac{(N-K)\sum_{i=1}^{K}n_{i}{\left({\bar{Z}}_{i}-\bar{Z}\right)}^{2}}{(K-1)\sum_{i=1}^{g}\sum_{s=1}^{n_{i}}n_{i}{\left(Z_{is}-{\bar{Z}}_{i}\right)}^{2}}∼F(v_{1},v_{2})$$ (20)

with null and alternative hypotheses $H_{0}:\sigma_{1}^{2}=\sigma_{2}^{2}=\cdots =\sigma_{K}^{2}$ and *H*_1_: “Not all variances are homogeneous” (i.e. $\sigma_{i}^{2}\neq \sigma_{j}^{2}$ for some *i*≠*j*,*i*,*j* = 1,2,…,*K*), here *v*_1_ = *K*−1, *v*_2_ = *N*−*K* are the degrees of freedom for the F-statistics, $N=\sum_{i=1}^{K}n_{i}$, *K* is the number of features in the network, *n*_*i*_ = 100 is the sample size, $Z_{is}=|Y_{i,s}-{\bar{Y}}_{i}|$, where ${\bar{Y}}_{i}$ is the mean of the sample values *Y*_*i*,*s*_ of centrality *Y*_*i*_ (*i* = 1,2,…,*K*, *s* = 1,…,100). Substitute the centrality values into the F statistics, if $F\in \left(F_{1-\frac{\theta }{2}}(v_{1},v_{2}),F_{\frac{\theta }{2}}(v_{1},v_{2})\right)$, *H*_0_ is accepted and all variances are deemed to be homogeneous, otherwise, i.e. $F\notin \left(F_{1-\frac{\theta }{2}}(v_{1},v_{2}),F_{\frac{\theta }{2}}(v_{1},v_{2})\right)$, *H*_1_ is accepted (*P*<*θ*), the variances are deemed non-homogeneous. We have tested the variance homogeneity for significant levels *θ*∈{0.25, 0.1, 0.05, 0.025, 0.01, 0.005}, results of all *θ* values indicate that the variances are non-homogeneous. This is possible, because for the normalized centrality values, the structural independent features (common features for all structural classes) attain persistent high or low centralities for all random permutations, these will get smaller variances than others. Since we aim to compare the significant differences between the features, the centrality differences will be sensitive to the significance tests, thus we do not do data-transformations to fit for the variance homogeneity requirement for general hypothesis corrections, but use pairwise Welch T-tests which are independent of the homogeneity of variances to detect the significant differences between the features. Since we focus on the centrality differences (i.e. high and low inferences) rather than their equalities, we use the unilateral hypotheses in the Welch T-tests.

For a network of K features (nodes), we use the hypotheses *H*_0_: *μ*_*i*_≤*μ*_*j*_, *H*_1_: *μ*_*i*_>*μ*_*j*_ and $H_{0}^{\prime }:\mu_{i}\geq \mu_{j},$ *H*_2_: *μ*_*i*_<*μ*_*j*_ to test whether the centrality *Y*_*i*_ is significantly higher (hypotheses *H*_0_, *H*_1_) or lower (hypotheses $H_{0}^{\prime },H_{2}$) than *Y*_*j*_ (*i*≠*j*, *i*,*j* = 1,2,…,*K*). The Welch T-tests use T-statistics [53]:

$$T=\frac{{\bar{Y}}_{i}-{\bar{Y}}_{j}}{\sqrt{\frac{S_{i}^{2}}{n_{i}}+\frac{S_{j}^{2}}{n_{j}}}}∼T(v),$$ (21)

with the $v=\frac{{\left(S_{i}^{2}/n_{i}+S_{j}^{2}/n_{j}\right)}^{2}}{\frac{{\left(S_{i}^{2}/n_{i}\right)}^{2}}{n_{i}-1}+\frac{{\left(S_{j}^{2}/n_{j}\right)}^{2}}{n_{j}-1}}$ degree of freedom, *Y*_*i*_ and *Y*_*j*_ represent the centralities of features *i* and *j*, $S_{i}^{2},S_{j}^{2}$ are the usual estimates of sample variance of *Y*_*i*_ and *Y*_*j*_, *n*_*i*_ = *n*_*j*_ = 100 are the sample sizes of *Y*_*i*_ and *Y*_*j*_ (*i*≠*j*,*i*,*j* = 1,2,…,*K*). Substitute in the centrality values, if *T*≥*T*_*θ*_(*v*) (*P*<*θ*), then *H*_1_: *μ*_*i*_>*μ*_*j*_ is accepted and the centrality *Y*_*i*_ is deemed significantly higher than the centrality *Y*_*j*_; otherwise, *H*_0_: *μ*_*i*_≤*μ*_*j*_ is accepted, we need to further check $H_{0}^{\prime }$ and *H*_2_. When *H*_0_ is accepted, we check if *T*≤−*T*_*θ*_(*v*) (*P*<*θ*), then *H*_2_: *μ*_*i*_<*μ*_*j*_ is accepted, the centrality *Y*_*i*_ is deemed significantly lower than the centrality *Y*_*j*_; otherwise, $H_{0}^{\prime }:\mu_{i}\geq \mu_{j}$ is accepted, both *H*_0_: *μ*_*i*_≤*μ*_*j*_ and $H_{0}^{\prime }:\mu_{i}\geq \mu_{j}$ hold, the centralities *Y*_*i*_ and *Y*_*j*_ are deemed to have no significant differences. We use Welch T-tests between each pair of features, all features are thus ordered by the significance of the centralities. We use these ordered results to discuss the significant high and low centralities in our analysis. We use standard significance levels *θ*∈{0.25, 0.1, 0.05, 0.025, 0.01, 0.005} (as in any statistical text books) for the Welch T-tests, where all *θ* values get similar ordered results. However, as *θ* decreases, the rejection regions of *H*_0_ and $H_{0}^{\prime }$ become narrow, thus less significant differences will be detected for smaller *θ*. Larger *θ* values such as *θ* = 0.25, 0.1, 0.05 may get wider rejection regions for the null hypothesis, which result in more significant differences to be identified, and thus better ordered results. To balance for both the significant differences and the proportions of significances, we consider all *θ*∈{0.25, 0.1, 0.05, 0.025, 0.01, 0.005}.

## Results

**Fig 1:**
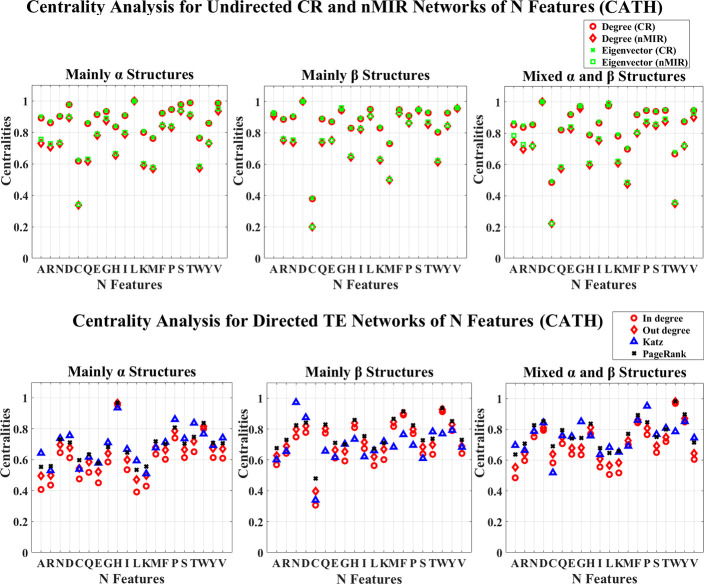
Centrality analysis for the networks of N features (CATH). This figure shows the centrality results for the networks of N features (CATH data). The normalized centralities are plotted against the features (represented by the amino acid abbreviations). In the CR and nMIR networks, the red curves represent the degree centralities, while the green curves represent the eigenvector centralities. In the TE networks, the red curves present the in and out degree centralities, the blue and black curves represent the Katz and PageRank centralities.

**Fig 2:**
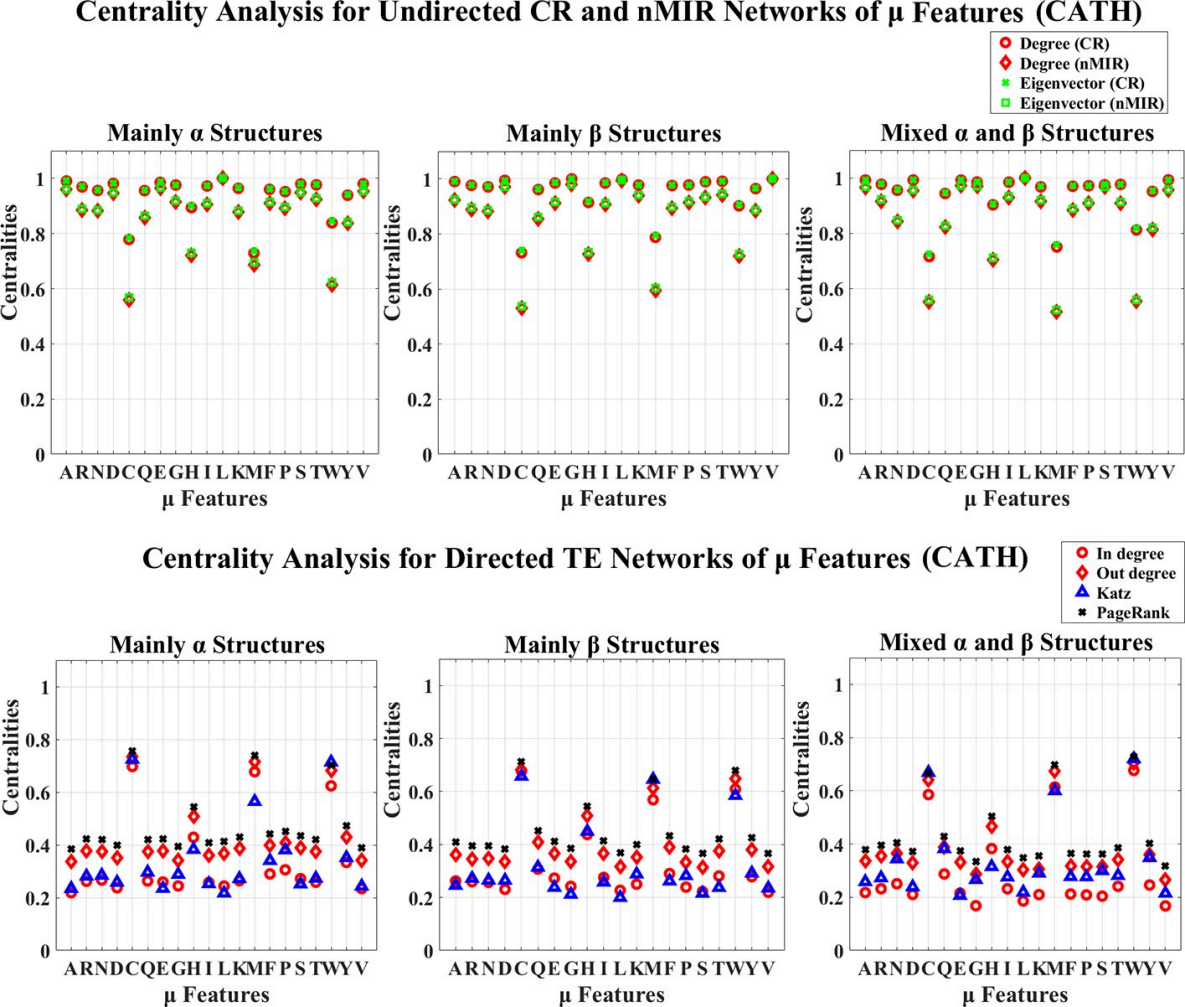
**Centrality analysis for the networks of N features (SCOP).** This figure shows the centrality results for the networks of the N features (SCOP data).

**Fig 3:**
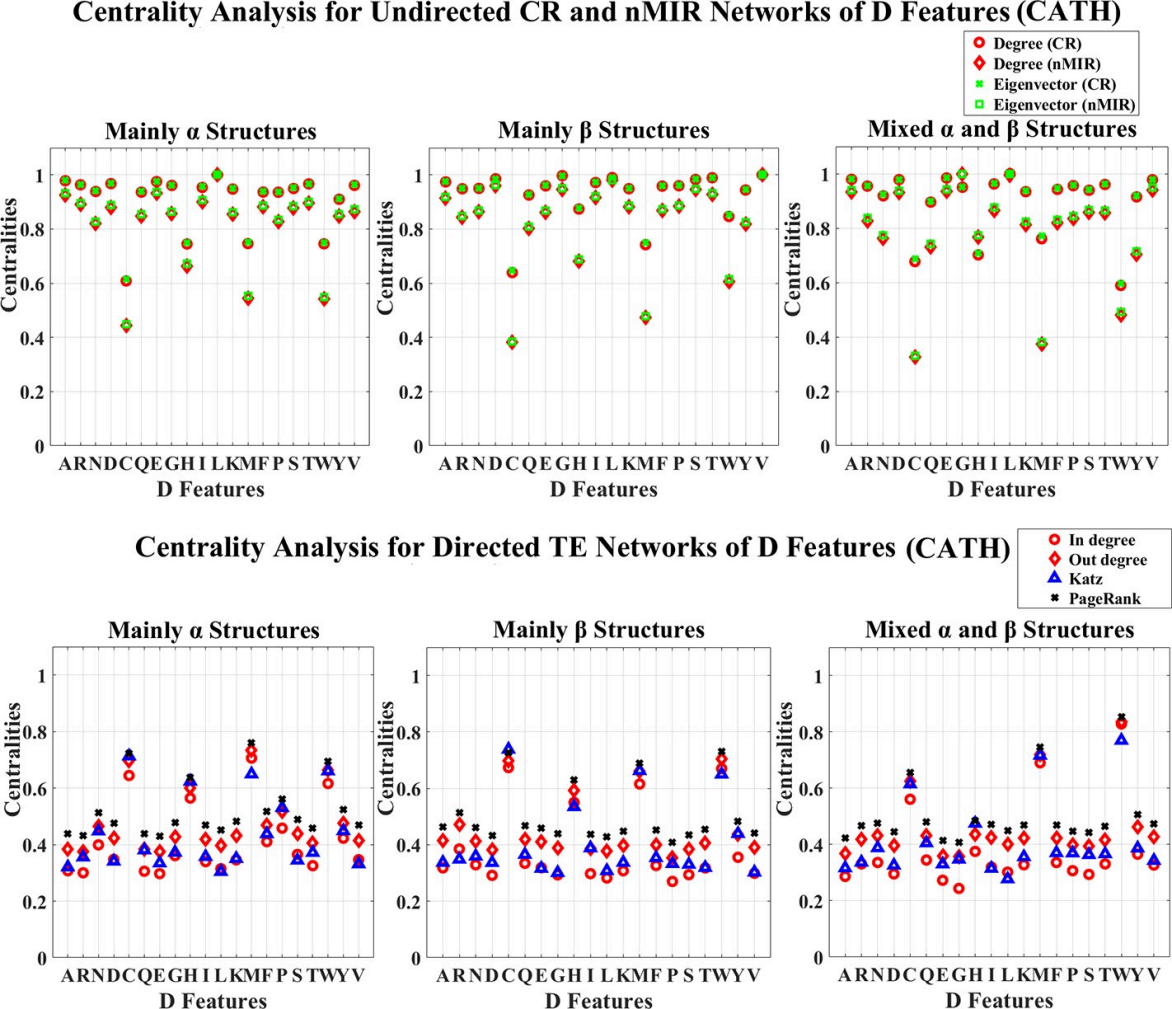
Centrality analysis for the networks of *μ* features (CATH). This figure shows the centrality results for the networks of *μ* features (CATH data).

**Fig 4:**
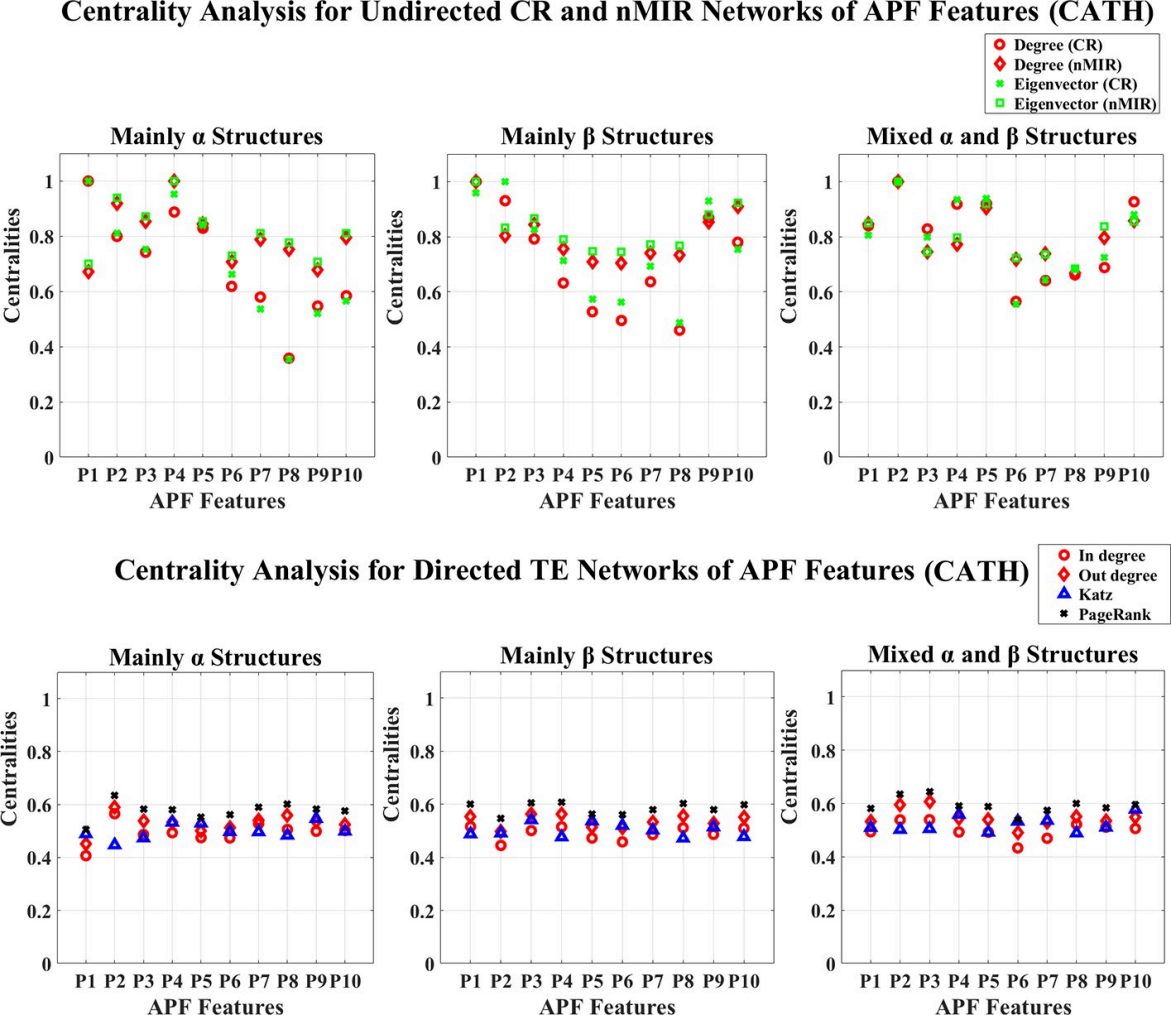
Centrality analysis for the networks of *μ* features (SCOP). This figure shows the centrality results for the networks of *μ* features (SCOP data).

**Fig 5:**
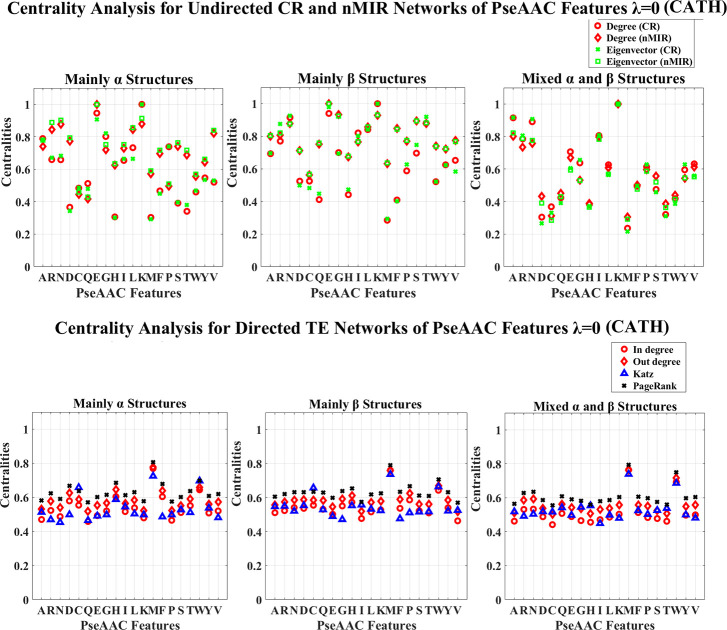
Centrality analysis for the networks of D features (CATH). This figure shows the centrality results for the networks of D features (CATH data).

**Fig 6:**
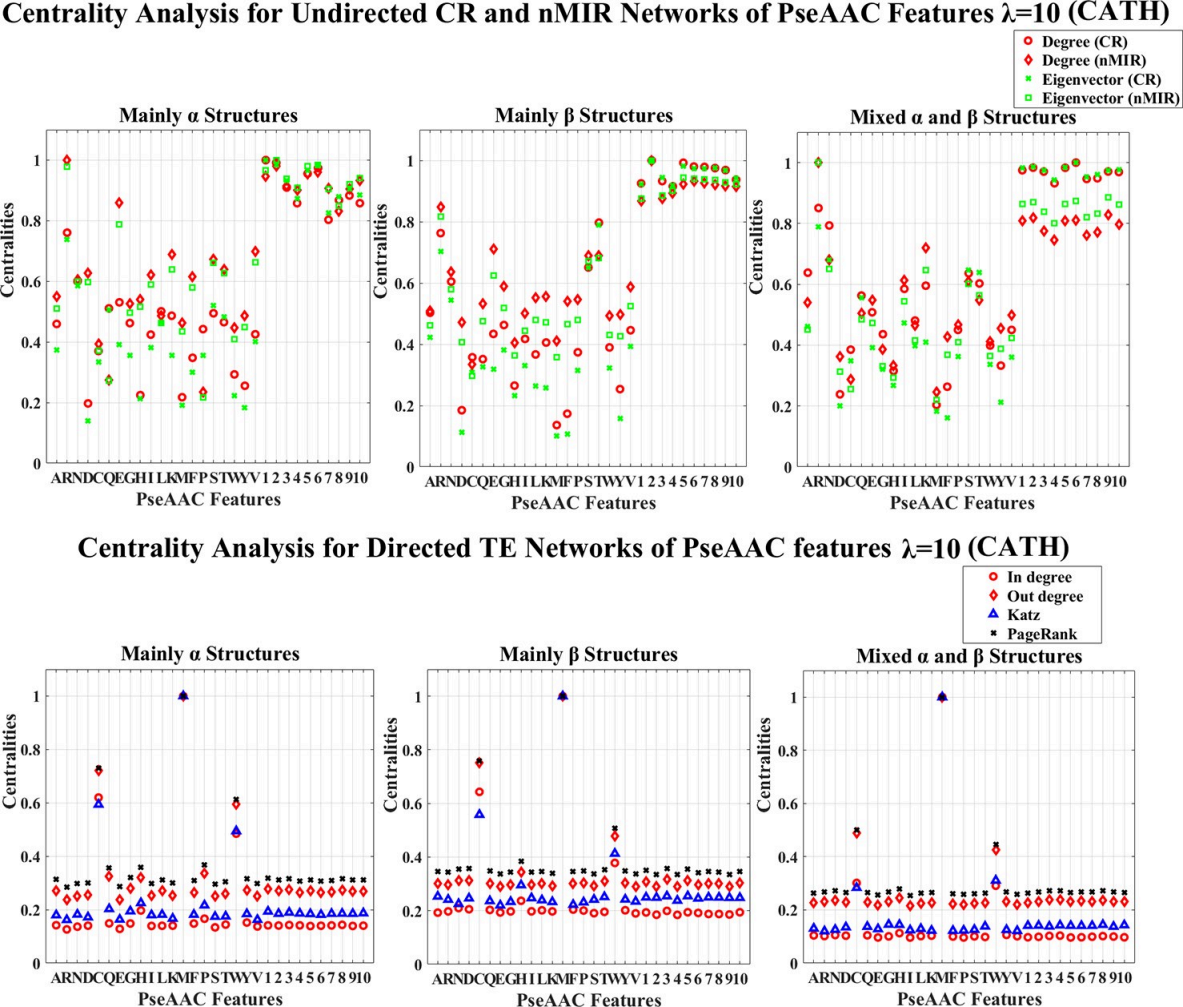
Centrality analysis for the networks of D features (SCOP). This figure shows the centrality results for the networks of D features (SCOP data).

**Fig 7:**
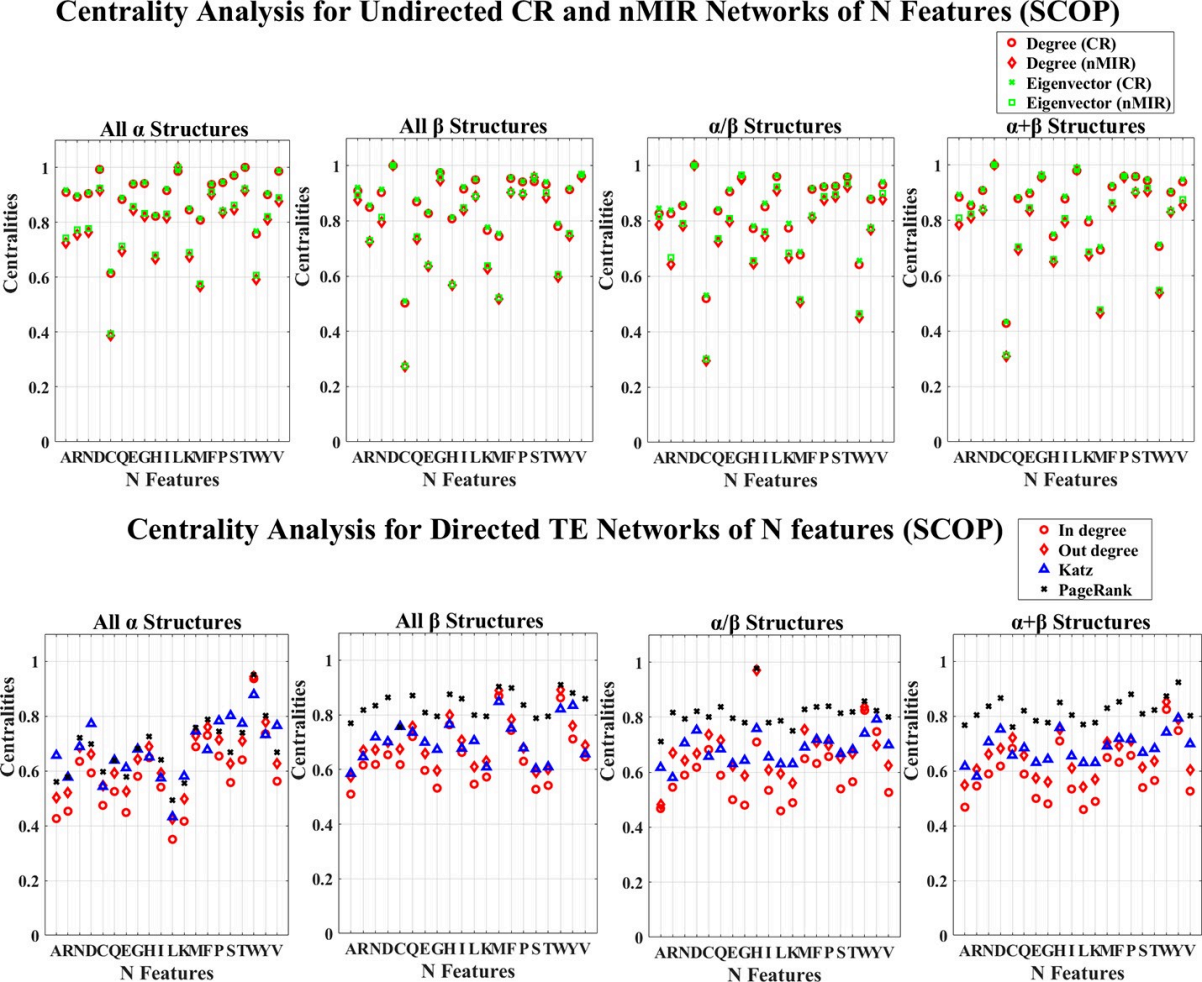
Centrality analysis for the networks of APF features (CATH). This figure shows the centrality results for the networks of APF features (CATH data). The normalized centralities are plotted against the features (represented by the indices of the properties as listed in S2 Table).

**Fig 8:**
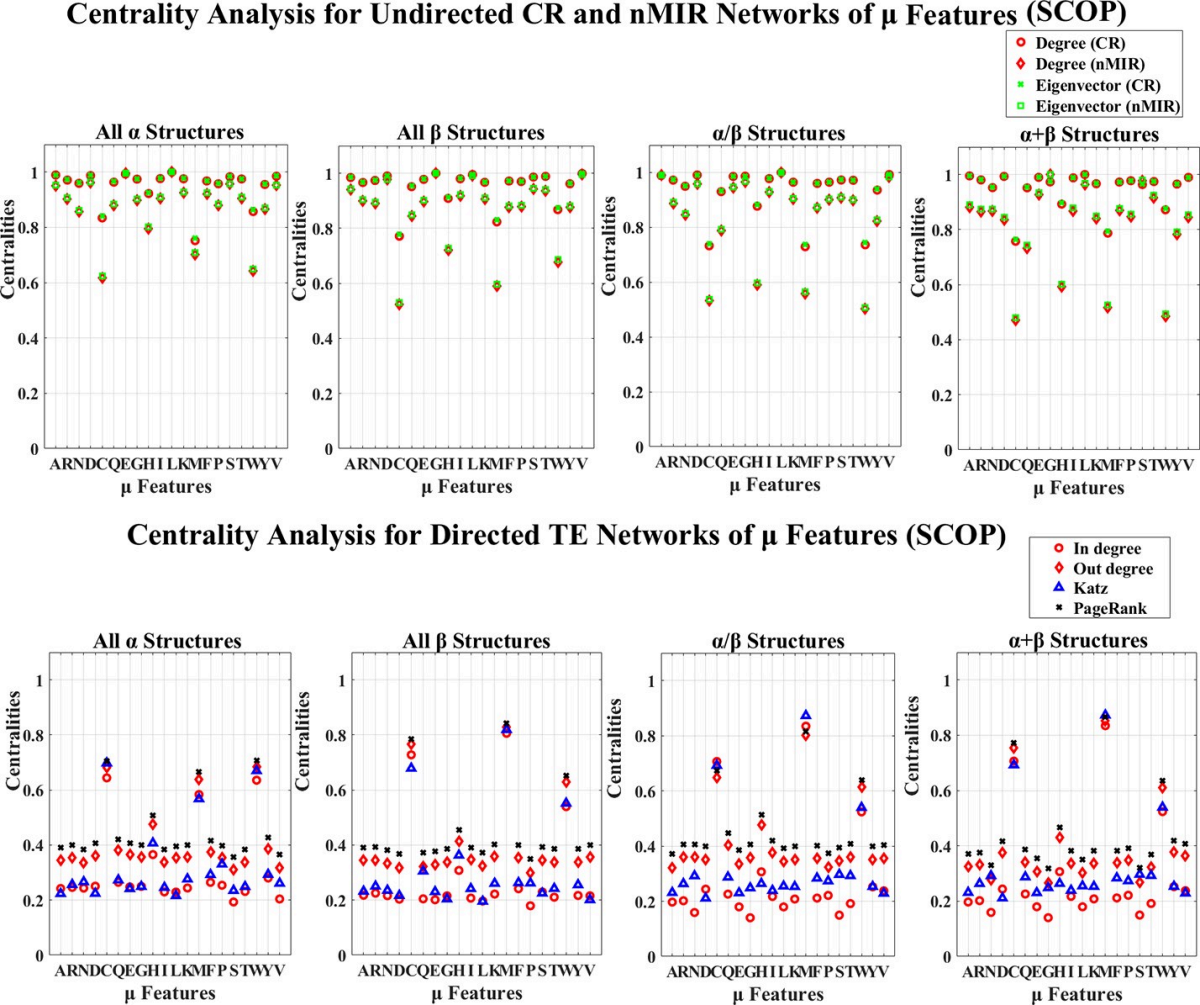
Centrality analysis for the networks of APF features (SCOP). This figure shows the centrality results for the networks of APF features (SCOP data).

**Fig 9:**
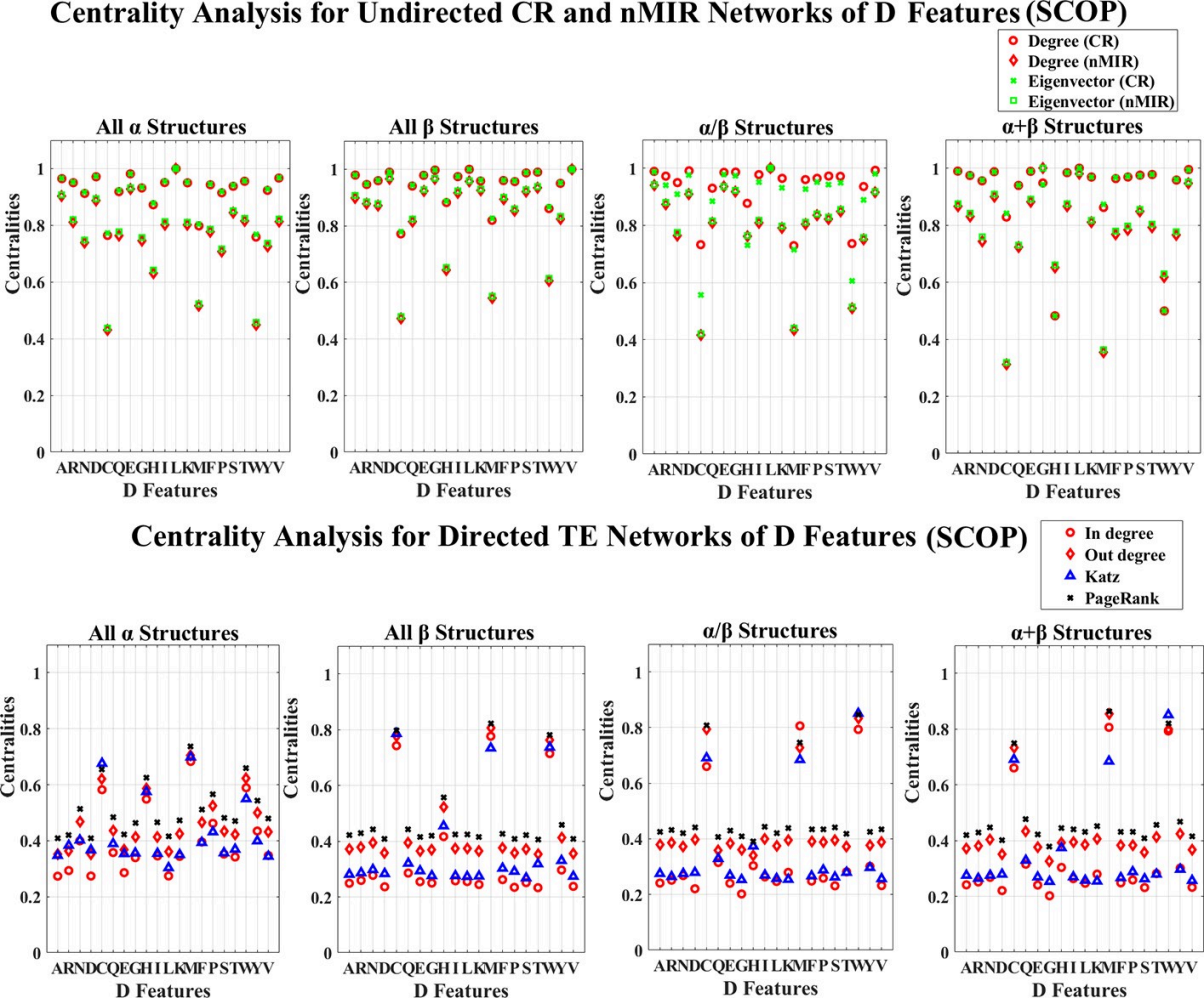
Centrality analysis for the networks of PseAAC features with *λ* = 0 (CATH). This figure shows the centrality results for the undirected CR and nMIR networks (upper plots) and the directed TE networks (bottom plots) for the PseAAC features with *λ* = 0 (CATH data).

**Fig 10:**
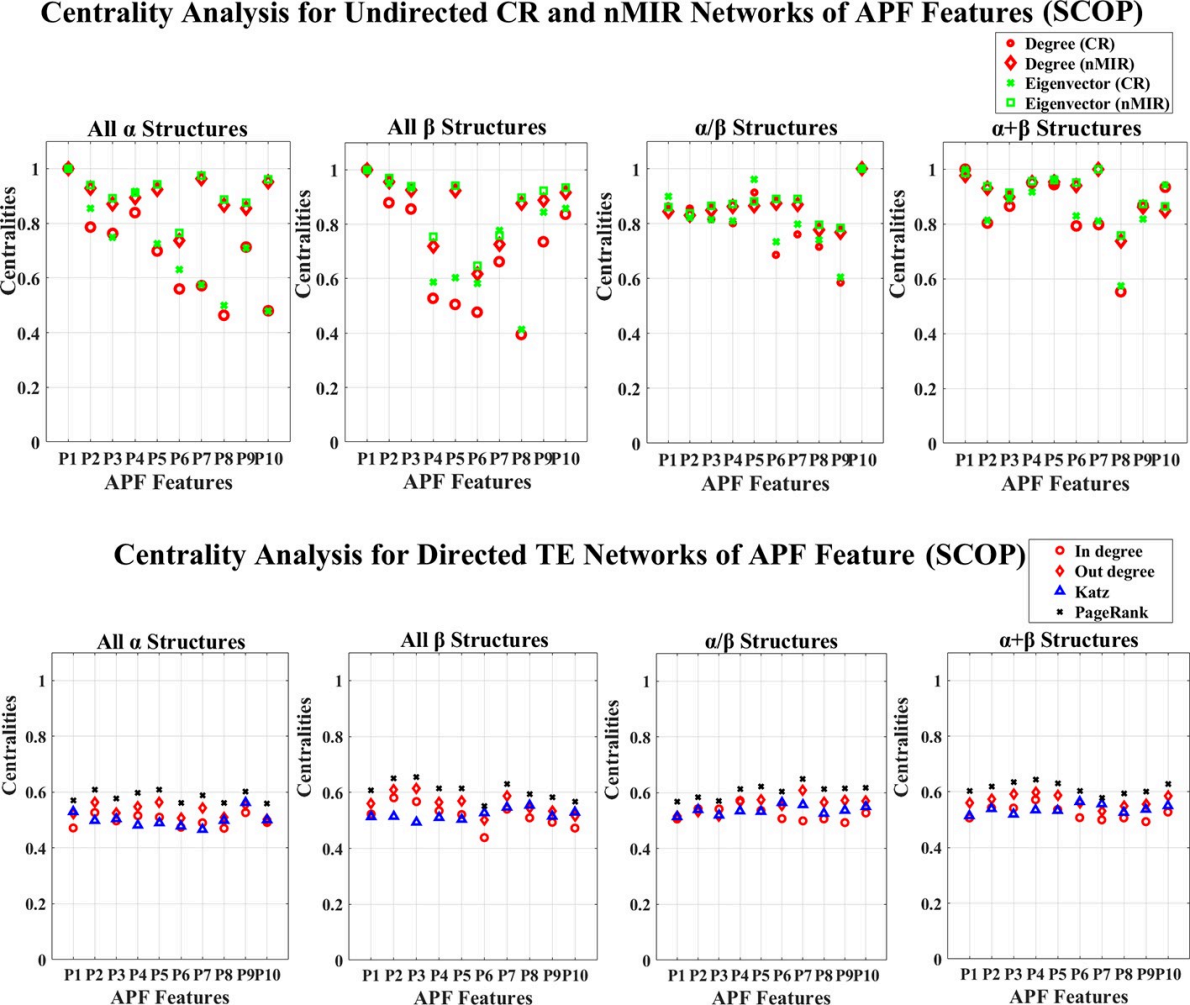
Centrality analysis for the networks of PseAAC features with *λ* = 0 (SCOP). This figure shows the centrality results for the undirected CR and nMIR networks (upper plots) and the directed TE networks (bottom plots) for the PseAAC features with *λ* = 0 (SCOP data).

**Fig 11:**
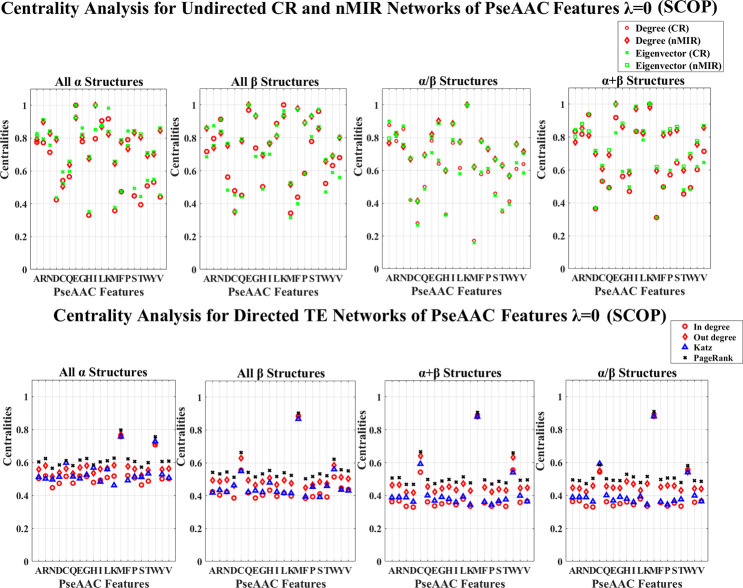
Centrality analysis for the networks of PseAAC features with *λ* = 10 (CATH). This figure shows the centrality results for the networks of PseAAC features with *λ* = 10 (CATH data). The normalized centralities are plotted against the features (represented by the amino acid abbreviations and the indices of the *λ*-tier correlations).

**Fig 12:**
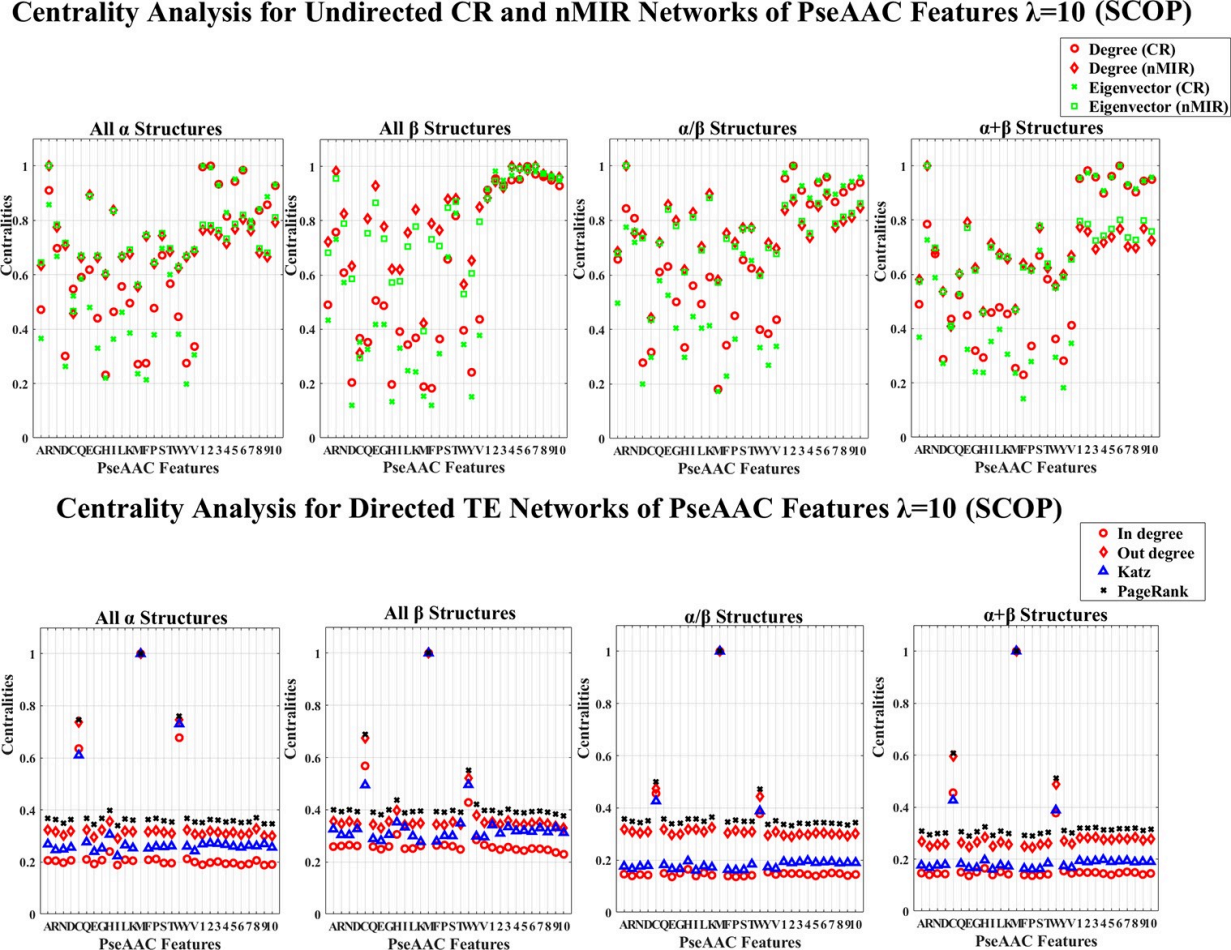
Centrality analysis for the networks of PseAAC features with *λ* = 10 (SCOP). This figure shows the centrality results for the networks of PseAAC features with *λ* = 10 (SCOP data).

We use the 30% similarity representative protein sequences in the entire CATH and SCOP databases to perform the analysis. The CATH data contains 8321 proteins, each of the top hierarchical classes contain 1673 (mainly *α* class), 1772 (mainly *β* class), and 4876 (mixed *α* and *β* class) proteins. The SCOP data contains 4836 proteins, and the four top hierarchical classes separately contain 960 (all *α* class), 1030 (all *β* class), 1490 (*α*/*β* class), 1356 (*α*+*β* class) proteins. The PDB IDs of the CATH and SCOP data are shown in the S1 and S2 Datasets. We use the NV, APF and PseAAC to extract protein sequence features for each of the structure classes, and use CR, nMIR and TE relationship matrices to construct weighted networks to compute the normalized centralities for the different networks. The centrality results are shown in S3 and S4 Datasets, and the averaged standard deviation results for the normalized centralities are shown in Tables 1–4. In these Tables, the standard deviations are low, especially for undirected networks. This proves the robustness of the results. The normalized centrality results are shown in Figs 1–12. We use pairwise Welch T-tests to test the significant differences between the centralities, as *θ* varies in {0.25, 0.1, 0.05, 0.025, 0.01, 0.005}, all *θ* values present similar ordered results, but as *θ* decreases more features are judged with no significant differences, larger *θ* values such as *θ*∈{0.25, 0.1, 0.05} identify more significant differences hence better ordered results, smaller *θ* values get more rigorous rejection regions for the null hypothesis, thus better identification for the true significance. In this paper, we consider significant centralities for all *θ* values, where majority of the results hold for the most rigid significance level *θ* = 0.005 (*P*<0.005), except for a few cases the results hold for *θ*≥0.01. Nevertheless, all the significance results hold for *θ* = 0.05 (*P*<0.05). In the Results and Discussion sections, the significant high and low centralities are referred to the significant results with *θ* = 0.05 (*P*<0.05) according to the pairwise Welch T-tests. Sample results of the centrality orders with *θ* = 0.05 are shown in S2 and S3 Texts, the complete results for all *θ* values are shown in S5 Dataset.

Figs 1 and 2 show the centrality results for the networks of N features. In the undirected CR and nMIR networks (upper plots), all top hierarchical classes of CATH and SCOP show significant high centralities for the N features of Aspartic acid (D), Leucine (L), Valine (V), Serine (S), Threonine (T), but significant low centralities for Cystine (C), Methionine (M) and Tryptophan (W), Lysine (K), Histidine (H), particularly for Cystine (C), Methionine (M) and Tryptophan (W). These imply that all structural classes contain significant strong symmetric relations between the numbers of Aspartic acid (D), Leucine (L), Valine (V), Serine (S), Threonine (T) and other features, while the numbers of Cystine (C), Methionine (M), Tryptophan (W), Lysine (K), Histidine (H) show significant weak symmetric relations with other features. These features are common for all protein structural classes that may not have great influences in differentiating the different types of structures.

Except for these commonalities, the different structural classes have different preferences of the significant centralities. The *α* structures (mainly *α* and all *α* classes) present significant low centralities for Glutarnine (Q), while the *β* structures (mainly *β* and all *β* classes) present significant high centralities for Phenylalanine (F), Glycine (G). The mixed structural classes (mixed *α* and *β* class and the *α*/*β*, *α*+*β* classes) show significant high centralities for Glycine (G), while the *α*/*β* class presents significant low centralities for Arginine (R) in the nMIR networks, the *α*+*β* class presents significant high centralities for Proline (P), but significant low centralities for Glutarnine (Q) in nMIR networks. These imply that the *α* structures contain significant weak symmetric feature relations for the numbers of Glutarnine (Q), while the *β* structures prefer significant strong symmetric feature relations for the numbers of Phenylalanine (F), Glycine (G), the mixed structures admit significant strong symmetric relations for the numbers of Glycine (G). Moreover, the *α*/*β* class prefers significant weak symmetric nonlinear relations with the numbers of Arginine (R), while the *α*+*β* class prefers significant strong symmetric relations for the numbers of Proline (P), but significant weak symmetric nonlinear relations with the numbers of Glutarnine (Q). The mixed structures may contain significant features from either *α* or *β* structures.

In the directed networks of N features (bottom plots of Figs 1 and 2), all protein structural classes admit significant high centralities for the N features of Histidine (H) and Tryptophan (W), but significant low centralities for Lysine (K), Alanine (A), Leucine (L). The top hierarchical classes of CATH also admit significant high centralities for Asparagine (N), Aspartic acid (D). These imply that the strong asymmetric relations for the numbers of Tryptophan (W), Histidine (H), and weak asymmetric relations for the numbers of Lysine (K), Alanine (A), Leucine (L) are structural independent that may not have great influences in differentiating the different types of structures. The *α* structures (mainly *α* and all *α* classes) also admit significant high centralities for Proline (P), Threonine (T), but significant low centralities for Glutamic acid (E), Arginine (R). The *β* structures (mainly *β* and all *β* classes) admit significant high centralities for Methionine (M), Phenylalanine (F), Tyrosine (Y). The mainly *β* class also admits significant low centralities for Cystine (C), while the all *β* class admits significant weak centralities for Threonine (T), Glycine (G), Serine (S). The mixed structural classes show significant centrality preferences from both *α* and *β* structures. The mixed *α* and *β* class admit significant high centralities for Phenylalanine (F), Tyrosine (Y), Proline (P), but significant low centralities for Cystine (C), Isoleucine (I); the *α*/*β* class admits significant high centralities for Cystine (C), Glutarnine (Q), but significant low centralities for Glycine (G); the *α*+*β* class admits significant high centralities for Proline (P), Tyrosine (Y), but significant low centralities for Glutamic acid (E), Glycine (G). These imply that the *α* structures contain significant strong asymmetric relations for the numbers of Proline (P), Threonine (T) but significant weak asymmetric relations for Glutamic acid (E), Arginine (R). The *β* structures admit significant strong asymmetric relations for the numbers of Methionine (M), Phenylalanine (F), Tyrosine (Y), the mainly *β* class prefers significant weak asymmetric relations for Cystine (C), while the all *β* class prefers significant weak asymmetric relations for Glycine (G), Threonine (T), Serine (S). The mixed *α* and *β* class shows significant weak asymmetric relations for the numbers of Cystine (C), Isoleucine (I); the *α*/*β* shows significant strong asymmetric relations for Cystine (C), Glutarnine (Q) but significant weak asymmetric relations for Glycine (G); the *α*+*β* shows significant strong asymmetric relations for Proline (P), Tyrosine (Y), but significant weak asymmetric relations for Glutamic acid (E), Glycine (G).

The asymmetric relations are independent of the symmetric relations. For instance, the *α*/*β*, *α*+*β* classes show significant strong symmetric relations for the numbers of Glycine (G), but significant weak asymmetric relations for Glycine (G), which imply that the relations between the numbers of Glycine (G) and other amino acids are symmetric (probably deterministic) rather than asymmetric (probably non-deterministic).

Figs 3–6 show the results for the *μ* and D features. In these figures, all top hierarchical classes of CATH and SCOP show significant high centralities for the *μ* and D features of Alanine (A), Aspartic acid (D), Leucine (L), Serine (S), Threonine (T), Valine (V), but significant low centralities for Cystine (C), Methionine (M), Tryptophan (W), Histidine (H) in the undirected networks, and also significant high centralities for the *μ* and D features of Cystine (C), Histidine (H), Methionine (M), Tryptophan (W) in directed networks. These imply that the arrangement features of Cystine (C), Methionine (M), Tryptophan (W), Histidine (H) attain weak symmetric but strong asymmetric relations with other amino acids, while the arrangements of Alanine (A), Aspartic acid (D), Leucine (L), Serine (S), Threonine (T), Valine (V) attain significant strong symmetric relations with other amino acids. These significant feature relations are common for all top hierarchical classes of CATH and SCOP, which may not be critical in differentiating the different types of structures. The arrangement features of Serine (S) and Threonine (T) also show significant high centralities, but the magnitudes are significantly low than that of Alanine (A), Aspartic acid (D), Leucine (L), Valine (V). The *α* structures (mainly *α* and all *α* classes) also show significant strong (high centralities) symmetric relations for the arrangement features of Glutamic acid (E), while the *β* structures (mainly *β* and all *β* classes) also show significant strong symmetric relations for the arrangement features of Glycine (G). The mixed structural classes contain both *α* and *β* structures (mixed *α* and *β* class and *α*/*β*, *α*+*β* classes) show significant strong symmetric relations for the arrangement features of both Glutamic acid (E) and Glycine (G). The significant strong relations for the arrangements of Glutamic acid (E) and Glycine (G) are the key features for the *α* and *β* structures, respectively.

The centrality distributions for the APF networks are shown in Figs 7 and 8. In Figs 7 and 8, all top hierarchical classes of CATH and SCOP show significant high centralities for “Side-chain size” (*P*_2_), but significant low centralities for “Amino acid composition” (*P*_6_), “Flat extended preference” (*P*_7_), “Occurrence in *α* region” (*P*_8_) in CR networks, which imply that all top hierarchical classes of CATH and SCOP admit significant strong symmetric linear relations for “Side-chain size” (*P*_2_), but weak symmetric linear relations for “Amino acid composition” (*P*_6_), “Flat extended preference” (*P*_7_) and “Occurrence in *α* region” (*P*_8_). All these features are structural independent that are common for all top hierarchical classes of CATH and SCOP. However, there are also significant differences between the different protein structural classes. The *α* structures (mainly *α* and all *α* classes) show significant strong (high centralities) symmetric relations for “Side-chain size” (*P*_2_), “Extended structure preference” (*P*_3_), “Hydrophobicity” (*P*_4_), and strong symmetric linear relations for “Alpha-helix/bend preference” (*P*_1_), as well as significant weak symmetric linear relations for “Amino acid composition” (*P*_6_). The mainly *α* class also shows significant weak symmetric linear relations for “pk” (*P*_9_), the all *α* class shows significant strong symmetric nonlinear relations for “Alpha-helix/bend preference” (*P*_1_), “Flat extended preference” (*P*_7_), “Surrounding hydrophobicity in *β*–structure” (*P*_10_). The *β* structures (mainly *β* and all *β* classes) show significant strong symmetric relations for (*P*_1_), “Side-chain size” (*P*_2_), “Extended structure preference” (*P*_3_), “pk” (*P*_9_), “Surrounding hydrophobicity in *β*–structure” (*P*_10_), but weak symmetric relations for “Hydrophobicity” (*P*_4_), “Amino acid composition” (*P*_6_), “Occurrence in *α* region” (*P*_8_), and weak symmetric linear relations for “Double-bend preference” (*P*_5_). The big difference between the mainly *β* and all *β* classes is that, the symmetric nonlinear relations for “Double-bend preference” (*P*_5_) is significantly strong in all *β* class, but weak in the mainly *β* class. The mixed structural classes admit significant strong symmetric relations for “Double-bend preference” (*P*_5_), and strong symmetric linear relations for “Surrounding hydrophobicity in *β*–structure” (*P*_10_). The mixed *α* and *β* class also shows significant strong symmetric nonlinear relations for “Side-chain size” (*P*_2_), “Surrounding hydrophobicity in *β*–structure” (*P*_10_), and strong linear relations for “Hydrophobicity” (*P*_4_), but weak symmetric relations for “Amino acid composition” (*P*_6_), “Flat extended preference” (*P*_7_), “Occurrence in *α* region” (*P*_8_). The *α*/*β* class admits significant strong symmetric nonlinear relations for “Surrounding hydrophobicity in *β*−structure” (*P*_10_), but weak symmetric relations for “pk” (*P*_9_); the *α*+*β* class admits significant strong relations for “Hydrophobicity” (*P*_4_), and strong symmetric nonlinear relations for “Flat extended preference” (*P*_7_), but significant weak symmetric relations for “Occurrence in *α* region” (*P*_8_), and weak symmetric nonlinear relations for “Surrounding hydrophobicity in *β*−structure” (*P*_10_).

The mixed structural classes (mixed *α* and *β* class, *α*/*β*, *α*+*β*) show not only significant features for the *α* and *β* structures, but also special features for the “Double-bend preference” (*P*_5_). The “Double-bend preference” (*P*_5_) attain significant high centralities in the mixed structural class, but medium or even low centralities in the *α* or *β* structures. Unlike regular *α* or *β* structures, the “Double-bend” have conformations like chain reversals occurring over three residues [41], the “Double-bend preference” (*P*_5_) evaluates the normalized frequency of these double bends identified by the opposite signs of two successive dihedral angles, this is a key feature that well differentiate the mixed structural classes from the *α* or *β* structures.

In the directed networks of APF features, all structural classes show similar distribution for the centralities, but there are still differences between the different types of structures. By the Welch T-tests, the *α* structures show significant high centralities for “Side-chain size” (*P*_2_), the *β* structures show significant high centralities for “Extended structure preference” (*P*_3_). The mixed *α* and *β* class admits significant high centralities for both “Side-chain size” (*P*_2_) and “Extended structure preference” (*P*_3_). The *α*/*β* class admits significant high centralities for “Double-bend preference” (*P*_5_) and “Flat extended preference” (*P*_7_). The *α*+*β* class admits significant high centralities for “Hydrophobicity” (*P*_4_).

Figs 9–12 show the PseAAC feature networks with *λ* = 0 and *λ* = 10. Figs 9 and 10 present the centralities for the proportional composition of the 20 amino acids (*λ* = 0), while Figs 11 and 12 present the composition of amino acids normalized by weights from the sequence order effects (*λ* = 10). In Figs 11 and 12, the 10-tier correlations for the amino acid sequence order effects show high centralities (undirected networks) for all structural classes, which imply that the sequence order effects are important for all types of structures.

In Figs 9 and 10 (PseAAC, *λ* = 0), all top hierarchical classes of CATH and SCOP present significant high centralities for the PseAAC features of Lysine (K), but low centralities for Aspartic acid (D), Cystine (C), Glutarnine (Q), Histidine (H), Methionine (M), Tryptophan (W) in undirected networks, as well as significant high centralities for Methionine (M), Tryptophan (W) in directed networks. There are also significant high centralities for the PseAAC features of Asparagine (N) and Arginine (R) in undirected networks, but these centralities are significantly lower than the centralities of Lysine (K). Moreover, the all *β*, *α*/*β*, *α*+*β* classes of SCOP show significant high centralities for Cystine (C) in directed networks. These imply that significantly strong symmetric relations for the proportional composition of Lysine (K), Asparagine (N), Arginine (R), and the significant weak symmetric relations for the proportional composition of Aspartic acid (D), Cystine (C), Glutarnine (Q), Histidine (H), Methionine (M), as well the significant the strong asymmetric relations for proportional composition of Methionine (M) and Tryptophan (W) are the common features for all structural classes.

For the undirected networks of PseAAC (*λ* = 0), there are also significant high centralities for the PseAAC features of Glutamic acid (E) and Leucine (L) in both *α* and *β* structures. The *α* structures (mainly *α* and all *α* classes) admit significant low centralities for Threonine (T), and significant high centralities for Glycine (G) in CR networks, and for Valine (V) in nMIR networks. The mainly *α* class admits significant high centralities for Alanine (A), Proline (P) in CR networks, while the all *α* class admits significant high centralities for Isoleucine (I) in both CR and nMIR networks. The *β* structures (mainly *β* and all *β* classes) admit significant high centralities for Threonine (T) and Glycine (G) in both CR and nMIR networks, and for Serine (S) and Phenylalanine (F) in nMIR networks, but significant low centralities for Phenylalanine (F) in CR networks. The mixed structural classes (mixed *α* and *β* class, *α*/*β* and *α*+*β* classes) admit significant high centralities for Alanine (A), Asparagine (N), Isoleucine (I), Arginine (R), but significant low centralities for Threonine (T). These imply that the proportional compositions of Glutamic acid (E) and Leucine (L) attain strong symmetric relations in both *α* and *β* structures, and the strong symmetric relations for the proportional compositions of Glycine (G), Threonine (T), the strong nonlinear symmetric relations for Phenylalanine (F), Serine (S), are key features for the *β* structures. The significant weak and strong symmetric relations for the proportional compositions of Threonine (T) respectively in the *α* and *β* structures, is the big difference between the *α* and *β* structures. Additionally, the medium centralities for Aspartic acid (D) in nMIR networks but significant low centralities for Aspartic acid (D) in CR networks for the *α* structures, indicates that the proportional composition of Aspartic acid (D) attains intensive nonlinear rather than linear symmetric interactions with other amino acids in the *α* structures. This is different from the *β* structures, where in *β* structures, the proportional composition of Aspartic acid (D) has low centralities in both CR and nMIR networks.

In Figs 11 and 12 (PseAAC features with *λ* = 10), all top hierarchical classes of CATH and SCOP admit significant high centralities for the PseAAC features of Arginine (R), Serine (S), Threonine (T) in both CR and nMIR networks, and for Glutamic acid (E) (particularly in nMIR networks), Asparagine (N) (particularly in CR networks), but significant low centralities for Aspartic acid (D), Histidine (H), Methionine (M), Phenylalanine (F), Tyrosine (Y) in the CR networks, and significant low centralities for Cystine (C) in nMIR networks, as well as significant high centralities for Cystine (C), Methionine (M), Tryptophan (W) in the directed networks. In the undirected networks, the *α* structures (mainly *α* and all *α* classes) show significant high centralities for Valine (V) and Lysine (K) in nMIR networks. The mainly *α* class admits significant low centralities for Glutarnine (Q) in nMIR networks, while the all *α* class admits significant high centralities for Isoleucine (I) in nMIR networks. The *β* structures (mainly *β* and all *β* classes) present significant high centralities for Threonine (T), and for Valine (V) in nMIR networks, and for Alanine (A) in CR networks. The mainly *β* class admits significant high centralities for Glycine (G), while the all *β* class shows significant high centralities for Lysine (K). The big differences between the *α* and *β* structures are that, the *α* structures admit significant higher centralities for Threonine (T) than Serine (S), while *β* structures admit the opposite trends by the Welch T-tests (*P*<0.05). We also note that the centralities of Glycine (G) rank higher in the *β* structures than in the *α* structures. The mixed structural classes (mixed *α* and *β* class and the *α*/*β*, *α*+*β* classes) present significant high centralities for Lysine (K) and Isoleucine (I) in the nMIR networks, and for Alanine (A) in CR networks. We can see that, except for the common features for all structural classes, the strong symmetric interactions for the proportional compositions of Glutamic acid (E), Lysine (K), Arginine (R), Leucine (L), particularly for Glutamic acid (E), are the key similarities for the *α* and *β* structures. Moreover, the proportional compositions of Glycine (G), Threonine (T) are special features for *β* structures, and the different trends for the symmetric relations with Threonine (T) and Serine (S) is a key difference between the *α* and *β* structures.

## Discussion

In this study, we treat the protein universe as a complex system, where we use time series connectivity measures to model the relations between sequence features into networks, and use fundamental centrality measures and Welch T-test to identify significant features for the different types of protein structures. By performing the centrality analysis, we find both similarities and differences between the different protein structural classes. In our analysis, all top hierarchical classes of CATH and SCOP show strong symmetric relations for the numbers and arrangement features of Aspartic acid (D), Leucine (L), Serine (S), Threonine (T), Valine (V), and for the proportional compositions of Arginine (R), Lysine (K), Serine (S), Threonine (T), Glutamic acid (E), Asparagine (N), the arrangement features of Alanine (A) (non-polar), as well as the physical property “Side-chain size” (*P*_2_). These strong symmetric probably deterministic relations are common for all structural classes of proteins. Except for these strong relations, there are also weak symmetric relations for the composition and arrangements of Cystine (C), Histidine (H), Methionine (M), Tryptophan (W), and weak symmetric linear relations for the proportional compositions of Aspartic acid (D), Glutarnine (Q), Phenylalanine (F), Tyrosine (Y) and physical properties “Amino acid composition” (*P*_6_), “Flat extended preference” (*P*_7_) and “Occurrence in *α* region” (*P*_8_). Moreover, all structural classes also admit strong asymmetric relations for the composition and arrangement features of Cystine (C), Methionine (M), Tryptophan (W), and the arrangement features of Histidine (H), but weak asymmetric relations for the composition numbers of Lysine (K), Alanine (A), Leucine (L), which indicate that these features are highly interactive with other features, and these asymmetric interactions may probably be non-deterministic interactions. All these common features significant for all top hierarchical classes of CATH and SCOP might be the structural independent features that may not have critical influential in encoding the different types of structures.

The different protein 3D structural classes also show different feature preferences. The *α* structures prefer significant strong symmetric relations for the proportional compositions of Isoleucine (I), Glutamic acid (E), Leucine (L), the arrangement features of Glutamic acid (E), and physical properties “Side-chain size” (*P*_2_), “Extended structure preference” (*P*_3_), “Hydrophobicity” (*P*_4_), and strong symmetric linear relations with “Alpha-helix/bend preference” (*P*_1_) and nonlinear relations with the proportional compositions of Valine (V), and weak symmetric relations for the composition numbers of Glutarnine (Q) and the proportional compositions of Threonine (T), “Amino acid composition” (*P*_6_), and strong asymmetric relations for the numbers of Proline (P), Threonine (T). We may suggest that these significant features may have great influences in encoding the *α* structures.

The *β* structures prefer strong symmetric relations for the proportional compositions of Glutamic acid (E), Leucine (L), Threonine (T), Glycine (G), the compositions and arrangement features of Glycine (G), and the composition numbers of Phenylalanine (F), and physical properties “Alpha-helix/bend preference” (*P*_1_), “Side-chain size” (*P*_2_), “Extended structure preference” (*P*_3_), “pk” (*P*_9_), “Surrounding hydrophobicity in *β* structures” (*P*_10_), and strong symmetric nonlinear relations with the proportional compositions of Phenylalanine (F), Valine (V), and weak symmetric relations for the proportional compositions of Aspartic acid (D), physical properties “Hydrophobicity” (*P*_4_), “Amino acid composition” (*P*_6_), “Occurrence in *α* region” (*P*_8_), and weak symmetric linear relations with “Double-bend preference” (*P*_5_), as well as strong asymmetric relations for the composition numbers of Methionine (M), Phenylalanine (F), Tyrosine (Y). These imply that the *β* structures prefer strong deterministic (symmetric) relations with the features of Asparagine (N), Glycine (G), Serine (S), Threonine (T), and “Alpha-helix/bend preference” (*P*_1_), “pk” (polarity parameters of solutes with certain degree of dissociation in aqueous solution) (*P*_9_), “Surrounding hydrophobicity in *β* structures” (*P*_10_), but weak deterministic relations with “Amino acid composition” (*P*_6_). We may suggest that these significant features are influential in encoding the *β* structures, which make senses, because the physical properties such as the “Surrounding hydrophobicity in *β* structures” (*P*_10_) is a set of hydrophobic indices regarding the *β*−structures [44], which should have critical influences in *β* structures. Particularly, a key difference between the *α* and *β* structures is that the *β* structures prefer weak symmetric relations for “Hydrophobicity” (*P*_4_) but strong symmetric interactions for Threonine (T), while the *α* structures present the opposite trends for these features.

The mixed hierarchical classes show strong symmetric relations for the arrangements of Glutamic acid (E), the composition and arrangements of Glycine (G), the proportional compositions of Alanine (A), Arginine (R), Isoleucine (I), Asparagine (N), and physical properties “Hydrophobicity” (*P*_4_), “Double-bend preference” (*P*_5_), and significant strong symmetric nonlinear relations for “Surrounding hydrophobicity” (*P*_10_), but weak symmetric relations for the proportional compositions of Threonine (T) and physical property “Occurrence in *α* region” (*P*_8_). The mixed *α* and *β* class (CATH) also shows significant strong symmetric relations for “Side-chain size” (*P*_2_), significant strong asymmetric interactions for the numbers of Phenylalanine (F), Tyrosine (Y), Proline (P), and significant weak asymmetric interactions for the composition numbers of Cystine (C), Isoleucine (I), Glycine (G). The *α*/*β* (SCOP) shows weak symmetric relations for “pK” (*P*_9_) and weak nonlinear relations with the numbers of Arginine (R), as well as significant strong asymmetric relations for the numbers of Proline (P), Tyrosine (Y), but significant weak asymmetric relations for the numbers of Glutamic acid (E), Glycine (G). The *α*+*β* class (SCOP) presents significant strong symmetric relations for the numbers of Proline (P) and nonlinear relations for “Flat extended preference” (*P*_7_), but significant weak symmetric relations for “Occurrence in *α* region” (*P*_8_) and nonlinear relations for Glutarnine (Q), as well as strong asymmetric relations for the numbers of Cystine (C), Glutarnine (Q), and significant weak asymmetric relations for the numbers of Glycine (G). Most of the significant features for the mixed structural classes are inherited from the *α* and *β* structures. However, the strong symmetric relations for the “Double-bend preference” (*P*_5_) is a key factor for the mixed structural classes rather than the *α* and *β* structures.

From this analysis, we find the key differences between the *α* and *β* structures are the significant relations for the features of Serine (S), Threonine (T), Phenylalanine (F), Glycine (G), Glutarnine (Q), “Hydrophobicity” (*P*_4_), “pk” (*P*_9_), “Surrounding hydrophobicity in *β* structures” (*P*_10_). The *α* structures prefer significant strong symmetric relations for the arrangements of Glutamic acid (E), and “Hydrophobicity” (*P*_4_), but significant weak symmetric relations for the compositions of Threonine (T), Glutarnine (Q) and significant weak symmetric linear relations for “pk” (*P*_9_), “Surrounding hydrophobicity in *β* structures” (*P*_10_); while the *β* structures prefer significant strong symmetric relations for the compositions of Threonine (T), the compositions and arrangements of Glycine (G), the numbers of Phenylalanine (F), “pk” (*P*_9_), “Surrounding hydrophobicity in *β* structures” (*P*_10_), but significant weak symmetric relations for “Hydrophobicity” (*P*_4_). Moreover, the *α* structures show significant stronger symmetric relations for Serine (S) than Threonine (T), while the *β* structures show an opposite trend for these features.

We should note that the different amino acid features have different meanings. Both N and PseAAC features indicate amino acid compositions, the former account the discrete numbers of amino acids, while the latter account the proportions of compositions. Amino acids with the same N features may not have the same PseAAC features, and vice versa. The *μ* and D features interpret the sequence arrangement of amino acids, which show similar trends in the centrality analysis. The PseAAC features with *λ* = 10 also account for the sequence order effects, where the proportional compositions of amino acids are normalized by a weight from the 10-tier correlations of the sequence order effects.

As to the connectivity measures, both CR and nMIR indicate symmetric probably deterministic relations, while TE indicates asymmetric and probably non-deterministic relations. For an instance of a system X, both CR and nMIR get value 1 (for the deterministic relations) between X and itself, while TE gets 0 for this deterministic relation [54–58]. For another instance of the non-deterministic interactions in linear autoregressive models [54–58], the series are highly interactive but none of them are totally determined by each other, TE will get high positive values on interactive directions, while CR and nMIR will get 0 on all these interactive directions. The interactions captured by significant high positive TE values are symmetric and non-deterministic. In fact, TE will be vanished for deterministic relations. These indicate that high symmetric relations captured by CR and nMIR may not correspond with high asymmetric relations (described by TE), and vice versa. These can be seen from our analysis that the Cystine (C), Methionine (M), Tryptophan (W) get weak symmetric relations in undirected networks, but strong asymmetric relations in directed networks. These imply that there exist strong probably non-deterministic relations between these and other features.

The CR and nMIR also get differences in the symmetric relations. CR indicates the symmetric linear relations, while nMIR presents “model-free” symmetric relations that are no matter linear or not. If relations get low CR but high nMIR values, these mean that these symmetric relations are probably nonlinear. For instances of the *β* structures, the N features of Phenylalanine (F) show low centralities in CR networks, but high centralities in nMIR networks. These indicate that there exist strong nonlinear rather than linear relations for the numbers of Phenylalanine (F) in *β* structures. These nonlinear relations are not weird in real-world biological systems.

In this study, we use network methods to analyze significant relations between protein sequence features. We managed to identify significant features and interactions preferred by each type of the protein 3D structures. From these results, we can further explore the sequential influences to deeper protein structural levels, and also develop new tools for future protein structural classifications and predictions by considering the significant features identified for the different protein 3D structures. This analysis approaches the protein structural studies from a new relationship and network prospect, where all measures are fundamental and efficient, and the methods are exemplary for future protein structural or functional studies, or even genetic studies on virus and bacteria by adjusting the sequence features to gene features.

## Conclusions

In this paper, we use relationship and network approaches to analyze the complicated relations between protein sequence features, where we find both similarities and differences in terms of the significant features between the different protein 3D structural classes. The methods and results of this study can also be used for future protein structural or functional analysis, or other related protein or genetic studies.

## Supporting information

S1 Table The names and classifications of the 20 amino acids. This table shows the classifications, names and abbreviation symbols for the 20 types of amino acids.
(DOCX)

S2 Table The 10 physical property factors of amino acids. This table shows the names and descripts of the 10 important physical properties of amino acids.
(DOCX)

S3 Table Average standard deviations of network centralities with different numbers of random permutations. This table shows the changes of the mean standard deviations over different numbers of random permutations. The Average standard deviations are computed by averaging the standard deviation values for the centralities obtained by different connectivity and centrality measures and over the different structural classes.
(DOCX)

S1 Text Definition of PseAAC features. This text shows the detailed definitions of the PseAAC features.
(DOCX)

S2 Text Centrality orders detected by the pairwise Welch T-test (CATH). This text shows the centrality orders of the CATH data. The centrality orders are detected by the pairwise Welch T-tests with significance levels *θ* = 0.05.
(DOCX)

S3 Text Centrality orders detected by the pairwise Welch T-test (SCOP). This text shows the centrality orders of the SCOP data. The centrality orders are detected by the pairwise Welch T-tests with significance levels *θ* = 0.05.
(DOCX)

S1 Fig TE matrices with different parameter choices. In this figure, the color matrices present the TE results computed with different embedding parameters. We can see that the different parameters of TE present similar results.
(TIF)

S1 Dataset PDB IDs for the CATH data. This file contains the PDB IDs for the 30% sequence similarity CATH data. The PDB IDs for the three protein structural classes are stored in the variables named ‘PID_A’, ‘PID_B’, ‘PID_M’.
(MAT)

S2 Dataset PDB IDs for the SCOP data. This file contains the PDB IDs for the 30% sequence similarity SCOP data. The PDB IDs for the four protein structural classes are stored in the variables named ‘PID_1’, ‘PID_2’, ‘PID_3’, ‘PID_4’.
(MAT)

S3 Dataset Centrality results for the CATH data. This file stores the centrality results for the CATH data.
(MAT)

S4 Dataset Centrality results for the SCOP data. This file stores the centrality results for the SCOP data.
(MAT)

S5 Dataset Datasets for the centrality orders. This file stores the centrality orders (by features) for the CATH and SCOP data. The data structures “CATH” and “SCOP” store the centrality orders by features, where ‘C1_ud’ and ‘C1_d’ store the results for the *α* structures in undirected and directed networks, respectively. The notations for the other structural classes are similarly defined. The “FeatureOrders” in deeper levels stores the centrality orders (descending order) by their significance. The “Scores” stores the scores of features (the numbers of features have significantly lower centralities than this feature) in descending order. Features with higher scores attain significantly higher centralities than features with lower scores, while features with the same scores admit no significant centrality differences by the Welch T-tests. The results are for all *θ*∈{0.25, 0.1, 0.05, 0.025, 0.01, 0.005}.
(MAT)
