# Supplementary material for: A protein structural study based on the centrality analysis of protein sequence feature networks
Source: PLoS One. 2021 Mar 29;16(3):e0248861. doi: 10.1371/journal.pone.0248861 (PMC8006989; doi:10.1371/journal.pone.0248861)
Supplement: S1 Table — This table shows the classifications, names and abbreviation symbols for the 20 types of amino acids. (DOCX) [file pone.0248861.s001.docx]

**S1 Table. The names and classifications of the 20 amino acids.** This table shows the classifications, names and abbreviation symbols for the 20 types of amino acids [1].

| **Classifications** | **Names of amino acids** | **Three-letter abbreviations** | **Symbols** |
| --- | --- | --- | --- |
| **Non-polar**  **amino acids** | Alanine | ALA | A |
|  | Valine | VAL | V |
|  | Leucine | LEU | L |
|  | Isoleucine | ILE | I |
|  | Phenylalanine | PHE | F |
|  | Tryptophan | TRP | W |
|  | Methionine | MET | M |
|  | Proline | PRO | P |
| **Uncharged**  **polar amino**  **acids** | Serine | SER | S |
|  | Threonine | THR | T |
|  | Tyrosine | TYR | Y |
|  | Glutarnine | GLN | Q |
|  | Asparagine | ASN | N |
|  | Cystine | CYS | C |
|  | Glycine | GLY | G |
| **Positively charged (alkaline) amino acids** | Arginine | ARG | R |
|  | Lysine | LYS | K |
|  | Histidine | HIS | H |
| **Negatively charged (acidic) amino acids** | Aspartic acid | ASP | D |
|  | Glutamic acid | GLU | E |

**References:**

1. Wang J, Wang Z, Tian X. Bioinformatics: Fundementals and applications. Tsinghua University Press. 2014.
