# Supplementary material for: A protein structural study based on the centrality analysis of protein sequence feature networks
Source: PLoS One. 2021 Mar 29;16(3):e0248861. doi: 10.1371/journal.pone.0248861 (PMC8006989; doi:10.1371/journal.pone.0248861)
Supplement: S2 Table — This table shows the names and descripts of the 10 important physical properties of amino acids. (DOCX) [file pone.0248861.s002.docx]

**S2 Table. The 10 physical property factors of amino acids.** In this table the first column are the indices for the 10 physical properties, the second column are the names of the physical properties [27, 38]. The values of the 10 physical properties for the 20 types of amino acids can be found in [38]. The first 4 property factors correspond essentially to single amino acid physical properties. The remaining 6 factors are superpositions of several physical properties and for convenience are identified by the property making the greatest contribution to the factor [38].

| **Index** | **Property factors** | **Descriptions** |
| --- | --- | --- |
| **P1** | $\alpha$-helix/bend preference | $\alpha$-helix or bend-structure preference-related, expresses the highly positive correlation for bend-structure preference and the highly negative correlation for $\alpha-$helix preference [38] |
| **P2** | Side-chain size | Bulk-related [38] |
| **P3** | Extended structure preference | $\beta-$structure preference related [38] |
| **P4** | Hydrophobicity | Hydrophobicity-related [38] |
| **P5** | Double-bend preference | Normalized frequency of double bend (identified by the opposite signs of two successive virtual-bond dihedral angles) [39] |
| **P6** | Amino acid composition | Average value of average amino acid compositions [40] |
| **P7** | Flat extended preference | Average relative fractional occurrence in extended structural regions $E_{0}$ [41] |
| **P8** | Occurrence in $\alpha$ region | Normalized frequency of $\alpha-$regions that are identified by backbone dihedral angles of amino acid residues [42] |
| **P9** | pK | pK-C, polarity parameters of solutes with certain degree of dissociation in aqueous solution [43] |
| **P10** | Surrounding hydrophobicity | Surrounding hydrophobicity in $\beta-$structure, a set of hydrophobic indices regarding $\beta-$structures [44] |
