# Supplementary material for: A protein structural study based on the centrality analysis of protein sequence feature networks
Source: PLoS One. 2021 Mar 29;16(3):e0248861. doi: 10.1371/journal.pone.0248861 (PMC8006989; doi:10.1371/journal.pone.0248861)
Supplement: S3 Table — This table shows the changes of the mean standard deviations over different numbers of random permutations. The Average standard deviations are computed by averaging the standard deviation values for the centralities obtained by different connectivity and centrality measures and over the different structural classes. (DOCX) [file pone.0248861.s003.docx]

**S3 Table. Average standard deviations of network centralities with different numbers of random permutations.** This table shows the changes of the mean standard deviations over different numbers of random permutations. The Average standard deviations are computed by averaging the standard deviation values for the centralities obtained by different centrality and connectivity measures and over the different structural classes.

| **Number of**  **Random**  **Permutations** | **Average Standard Deviations of Centralities** | | | |
| --- | --- | --- | --- | --- |
|  | **Undirected Networks (**$\boldsymbol{\times}\boldsymbol{10}^{\boldsymbol{-16}}$**)** | | **Directed Networks (**$\boldsymbol{\times}\boldsymbol{10}^{\boldsymbol{-1}}$**)** | |
|  | **CATH** | **SCOP** | **CATH** | **SCOP** |
| **10** | 4.02 | 3.21 | 1.57 | 1.28 |
| **20** | 4.58 | 3.73 | 1.67 | 1.23 |
| **30** | 5.21 | 4.41 | 1.679 | 1.21 |
| **40** | 5.53 | 4.76 | 1.70 | 1.19 |
| **50** | 6.12 | 5.45 | 1.70 | 1.18 |
| **60** | 7.13 | 6.50 | 1.70 | 1.44 |
| **70** | 7.67 | 7.09 | 1.70 | 1.56 |
| **80** | 7.84 | 7.25 | 1.70 | 1.61 |
| **90** | 8.47 | 7.93 | 1.70 | 1.66 |
| **100** | 9.39 | 9.01 | 1.70 | 1.69 |
