# Supplementary material for: A protein structural study based on the centrality analysis of protein sequence feature networks
Source: PLoS One. 2021 Mar 29;16(3):e0248861. doi: 10.1371/journal.pone.0248861 (PMC8006989; doi:10.1371/journal.pone.0248861)
Supplement: S1 Text — This text shows the detailed definitions of the PseAAC features. (DOCX) [file pone.0248861.s004.docx]

**S1 Text. Pseudo amino acid composition (PseAAC)**

PseAAC (Pseudo amino acid composition) is a 20 + $\lambda$ ($\lambda$ is a non-negative integer) dimensional real-vector represents the composition of the 20 types of amino acids and their sequence arrangements [1-6]. The essence of PseAAC is to include not only the main feature of amino acid composition, but also the information beyond amino acid composition [2]. The general PseAAC is defined by considering a protein chain of L amino acid residues [2]:

$R_{1}R_{2}R_{3}R_{5}R_{6}R_{7}\cdots R_{L}$ (1)

where the amino acid sequence order effect is approximated by a set of sequence order-correlated factors defined by [45]:

$\left\{ \begin{matrix} \begin{matrix} \theta_{1}=\frac{1}{L-1}\sum_{i=1}^{L-1} \Theta\left( R_{i},R_{i+1} \right) \\ \theta_{2}=\frac{1}{L-2}\sum_{i=1}^{L-2} \Theta(R_{i},R_{i+2}) \\ \theta_{3}=\frac{1}{L-3}\sum_{i=1}^{L-3} \Theta\left( R_{i},R_{i+3} \right),(\lambda<L) \end{matrix} \\ \cdots\\ \theta_{\lambda}=\frac{1}{L-\lambda}\sum_{i=1}^{L-\lambda} \Theta(R_{i},R_{i+\lambda}) \end{matrix} \right.$ (2)

where $\theta_{1}$ is the first-tier correlation factor that reflects the sequence order correlation between all the most contiguous residues along a protein chain,$\theta_{2}$ is the second-tier correlation factor that reflects the sequence order correlation between all the second most contiguous residues, and so forth. In Equation (2), the correlation function is defined by [2]

$\Theta\left( R_{i},R_{j} \right)=\frac{1}{3}\{\left[ H_{1}\left( R_{j} \right)-H_{1}\left( R_{i} \right) \right]^{2}+\left[ H_{2}\left( R_{j} \right)-H_{2}\left( R_{i} \right) \right]^{2}+{[M\left( R_{j} \right)-M\left( R_{i} \right)]}^{2}\}$ (3)

where $H_{1}\left( R_{i} \right)$, $H_{2}\left( R_{i} \right)$, and $M\left( R_{i} \right)$ are the hydrophobicity value, hydrophilicity value, and side-chain mass of the amino acid $R_{i}$, respectively, similarly the $H_{1}\left( R_{j} \right)$, $H_{2}\left( R_{j} \right)$, and $M\left( R_{j} \right)$ values for the amino acid $R_{j}$. The original hydophobicity, hydrphilicity, and side-chain mass values are all subjected to a standard conversion [2]:

$\left\{ \begin{matrix} H_{1}\left( i \right)=\frac{H_{1}^{0}\left( i \right)-\sum_{i=1}^{20} \frac{H_{1}^{0}(i)}{20}}{\sqrt{\frac{\sum_{i=1}^{20} {[H_{1}^{0}\left( i \right)-\sum_{i=1}^{20} \frac{H_{1}^{0}(i)}{20}]}^{2}}{20}}} \\ H_{2}\left( i \right)=\frac{xx-\sum_{i=1}^{20} \frac{H_{2}^{0}(i)}{20}}{\sqrt{\frac{\sum_{i=1}^{20} {[H_{2}^{0}\left( i \right)-\sum_{i=1}^{20} \frac{H_{2}^{0}(i)}{20}]}^{2}}{20}}} \\ M\left( i \right)=\frac{M^{0}\left( i \right)-\sum_{i=1}^{20} \frac{M^{0}(i)}{20}}{\sqrt{\frac{\sum_{i=1}^{20} {[M^{0}\left( i \right)-\sum_{i=1}^{20} \frac{M^{0}(i)}{20}]}^{2}}{20}}} \end{matrix} \right.$ (4)

before substituting into Equation (3), here $H_{1}^{0}\left( i \right)$ is the original [hydrophobicity value](http://www.csbio.sjtu.edu.cn/bioinf/PseAAC/ParaValue.htm) of the ith amino acid which can be found in [7], $H_{2}^{0}\left( i \right)$ is the corresponding original [hydrophilicity value](http://www.csbio.sjtu.edu.cn/bioinf/PseAAC/ParaValue.htm) which can be found in [8], and $M^{0}\left( i \right)$ the mass of the ith amino acid side chain that can be obtained from any biochemistry text book [2].

Without loss of generality, the numerical indices $1, 2, 3,\cdots, 20$ are used to represent the 20 native amino acids according to the alphabetical order of their single-letter codes: A, C, D, E, F, G, H, I, K, L, M, N, P, Q, R, S, T, V, W, Y. The data obtained by the standard conversion in Equation (4) will have a zero mean value and will remain unchanged if going through the same conversion procedure again.

From the definition of Equation (2), the amino acid arrangement of a protein is reflected by a set of sequence-correlation factors $\theta_{1}$,$\theta_{2},\theta_{3},\cdots,$ ,$\theta_{\lambda}$, by including the $\lambda$ sequence-correlation factors, the final (20 +$\lambda$)-dimensional vector of PseAAC is defined by [2] $X={[x_{1},\cdots,x_{20},x_{20+1},\cdots,x_{20+\lambda}]}^{T}$, where $x_{u}=\frac{f_{u}}{\sum_{i=1}^{20} f_{i}+\omega\sum_{j=1}^{\lambda} \theta_{j}}$ for $1\leq u\leq20$, and $x_{u}=\frac{\omega\theta_{u-20}}{\sum_{i=1}^{20} f_{i}+\omega\sum_{j=1}^{\lambda} \theta_{j}}$ for $20+1\leq u\leq20+\lambda$,$f_{u}$ is the normalized occurrence frequency of the 20 amino acids in the protein S [2], $\theta_{j}$ is the j-tier sequence correlation factor computed according to Equations (2-4) for the protein S, and $w>0$ is the weight factor for the sequence order effect [2]. The first 20 components of $X$ reflect the effects of amino acid composition, while the $20+1$ to $20+ \lambda$ components reflect the arrangements of amino acids.

The parameter $\lambda$ adjusts the sequence order effects to the composition of the 20 types of amino acids which should be no greater than the length of the protein sequence L. When $\lambda=0$, the PseAAC is the original occurrence frequency of the 20 types of amino acids. When $\lambda>0$, the first 20 components $x_{u}\left( 1\leq u\leq20 \right)$ are not the original proportional composition of the 20 amino acids but the composition effects modified with weighted terms of the sum of the $\lambda$-tier correlation term $\sum_{j=1}^{\lambda} \theta_{j}$ [45]. The optimum choice of parameter $\lambda$ can be tested by the Covariant Discriminant Algorithm (CDA) [2].

**References:**

1. Shen H, Chou K. PseAAC: A flexible web server for generating various kinds of protein pseudo amino acid composition. Analytical Biochemistry. 2008; 373: 386-388.

2. Chou KC. Prediction of protein cellular attributes using pseudo-amino-acid-composition. Proteins: Structure, Function, and Genetics. 2001; 43: 246-255.

3. Chou KC. Using amphiphilic pseudo amino acid composition to predict enzyme subfamily classes. Bioinformatics, 2005; 21: 10-19.

4. Chou KC, Cai YD. Prediction of membrane protein types by incorporating amphipathic effects. Journal of Chemical Information and Modeling. 2005; 45: 407-413.

5.Shen HB, Chou KC. Ensemble classifier for protein folding pattern recognition. Bioinformatics. 2006; 22: 1717-1722.

6. Chou KC. Prediction of Protein Subcellular Locations by Incorporating Quasi-Sequence-Order Effect. Biochemical and Biophysical Research Communications. 2000; 278: 477-483.

7. Tanford C. Contribution of hydrophobic interactions to the stability of the globular conformation of proteins. [Journal of the American Chemical Society](http://www.baidu.com/link?url=QGoduD_t2jR-0Kw44tv5XNrdrZ2_GYAiqWx6GtjRhywuTXMS2CqTQOAPH0k2RlaY). 1962; 84:4240-4274.

8. Hopp TP, Woods KR. Prediction of protein antigenic determinants form amino acid sequences. PNAS. 1981; 78: 3824-3828.
