# Supplementary material for: A protein structural study based on the centrality analysis of protein sequence feature networks
Source: PLoS One. 2021 Mar 29;16(3):e0248861. doi: 10.1371/journal.pone.0248861 (PMC8006989; doi:10.1371/journal.pone.0248861)
Supplement: S2 Text — This text shows the centrality orders of the CATH data. The centrality orders are detected by the pairwise Welch T-tests with significance levels θ = 0.05. (DOCX) [file pone.0248861.s005.docx]

| **Structural**  **classes** | **Measures** | | **Centrality orders for networks of N and**$\boldsymbol{\mu}$ **features (CATH)** | |
| --- | --- | --- | --- | --- |
|  |  |  | **N** | $\boldsymbol{\mu}$ |
| **Mainly** $\boldsymbol{\alpha}$ | **CR** | **D** | LTVSDPGFEINARYQHKWMC | LAEDVSTGIRKFNQPYHWCM |
|  |  | **E** | LTVSDPGFEINARQYHKWMC | LAEDVSTGIRKFQNPYHWCM |
|  | **nMIR** | **D** | LVSTDGFPIEAYNRHQKWMC | LEAVSDTGFIPRNKQYHMWC |
|  |  | **E** | LVSTDGFPIEAYNRHQKWMC | LEAVSDTGFIPRNKQYHMWC |
|  | **TE** | **DI** | HWPTNMYSDVFGIQCERKAL | CMWHYPFSNKQRTEIGLDVA |
|  |  | **DO** | HPTWDVNSFGYMIAQLECRK | CWMHPYFQGNRTKDISVEAL |
|  |  | **K** | HWPTNMDYVSFGIQCERKAL | CMWHYPFSKRENTQLIDGVA |
|  |  | **P** | HWPTNMDYVSFGIQCERKAL | CMWHYPFSKRENQTLIDGVA |
| **Mainly** $\boldsymbol{\beta}$ | **CR** | **D** | DVLFSGTYAPNIQREKHWMC | GVLDTASEIPKRFNYQHWMC |
|  |  | **E** | DVLFGSTYAPNIQREKHWMC | GVLDTASEIKPRFNYQHWMC |
|  | **nMIR** | **D** | DVGSFALPTYIRENQHKWMC | VLGDTKSAPEIFRYNQHWMC |
|  |  | **E** | DVGSFALTPYIRNEQHKWMC | VLGDTKSAPEIFRYNQHWMC |
|  | **TE** | **DI** | WFMHYDQPNIRVSTEKGALC | CWMHQFTYIEARNKGPDLSV |
|  |  | **DO** | NDYTWFHKGPMVLQRIESAC | CMWHQYKPRNDFIAETVSGL |
|  |  | **K** | WFMHYDQNPITVRSKEGALC | CWMHQFYTIEAKNRDGPVLS |
|  |  | **P** | WFMHYDQPNITVRSKEGALC | CWMHQFYTIEAKNRGDPLVS |
| **Mi10ed** $\boldsymbol{\alpha}$ **and** $\boldsymbol{\beta}$ | **CR** | **D** | DLGTVPSEFYINARQHKMWC | LVADEGIRTSPFKNYQHWMC |
|  |  | **E** | DLGVTPSEFYIANRQHKMWC | LVAEDGIRTSPFKNYQHWMC |
|  | **nMIR** | **D** | DLGVTPSEFIAYNRKHQMWC | LEGSAVDIRKTPFNQYHWCM |
|  |  | **E** | DLGVTPSEFAIRYNKHQMWC | LEGASVDIRKTPFNQYHWCM |
|  | **TE** | **DI** | WYFDHPNTQMSEGVRCIKLA | WMCHQNYTRIAEFDKPSLGV |
|  |  | **DO** | PFYGDTNWSQHEVAMLRKIC | WCMQYNHSKTFPIRGADLVE |
|  |  | **K** | WYFHDPNQTMSGEVRCIKLA | WMCHQNYRTAIEDFSPKLGV |
|  |  | **P** | WYFDPHNTQMSGEVRCIKLA | WMCHQNYRTAIEDFPSKLGV |

**Table A. Centrality orders for the networks of N and**$\boldsymbol{\mu}$ **features (CATH).** This table shows the centrality orders for the networks of N and$\mu$ features (CATH dataset) detected by pairwise Welch T-tests with significance level $\theta=0.05$. In the list of features (represented by abbreviations of amino acids) the left most features admit the significant highest ($P<0.05$) centralities among all features, while the right most features admit the significant lowest centralities among all features. The centralities are listed in descending orders (from left to right) according to the pairwise Welch T-tests. The full names and abbreviations of the 20 types of amino acids are presented in Supporting Information S1. The centrality orders for the other $\theta$ values can be found in Supporting Information S5 Dataset.

| **Structural**  **classes** | **Measures** | | **Centrality orders for networks of D and APF features (CATH)** | |
| --- | --- | --- | --- | --- |
|  |  |  | **D** | **APF** |
| **Mainly** $\boldsymbol{\alpha}$ | **CR** | **D** | LAEDTRVGISKNFQPYMWHC | $P_{1}, P_{4}, P_{5}, P_{2}, P_{3}, P_{6},P_{10}, P_{7}, P_{9}, P_{8}$ |
|  |  | **E** | LAEDTRVGISKNFQPYMWHC | $P_{1}, P_{4}, P_{5}, P_{2}, P_{3}, P_{6}, P_{10}, P_{7}, P_{9}, P_{8}$ |
|  | **nMIR** | **D** | LEAITRFDSVGKYQPNHMWC | $P_{4}, P_{2}, P_{3}, P_{5}, P_{10}, P_{7}, P_{8}, P_{6}, P_{9}, P_{1}$ |
|  |  | **E** | ELAITRDFSVGKYQPNHMWC | $P_{4}, P_{2}, P_{3}, P_{5}, P_{10}, P_{7}, P_{8}, P_{6}, P_{9}, P_{1}$ |
|  | **TE** | **DI** | MCWHPYFNSGDVKITLAQRE | $P_{2}, P_{7}, P_{8},P_{10}, P_{9}, P_{4},P_{3}, P_{5}, P_{6}, P_{1}$ |
|  |  | **DO** | CWMHPYNFQGTIRKSDEVAL | $P_{9},P_{4}, P_{5}, P_{10}, P_{6}, P_{7}, P_{1},P_{8},P_{3}, P_{2}$ |
|  |  | **K** | MCWHPYFNSKGDIVTLAQRE | $P_{2},P_{8}, P_{7}, P_{3},P_{9}, P_{4}, P_{10}, P_{6},P_{5},P_{1}$ |
|  |  | **P** | MCWHPYFNSKGDIVTLQARE | $P_{2},P_{8}, P_{7}, P_{9}, P_{3},P_{4}, P_{10}, P_{6},P_{5},P_{1}$ |
| **Mainly** $\boldsymbol{\beta}$ | **CR** | **D** | VGLTDSAIPEFNKRYQHWMC | $P_{1}, P_{2}, P_{9}, P_{3}, P_{10}, P_{7}, P_{4}, P_{5}, P_{6}, P_{8}$ |
|  |  | **E** | VGLTDSAIEPFNRKYQHWMC | $P_{2}, P_{1}, P_{9}, P_{3}, P_{10}, P_{4}, P_{7}, P_{5}, P_{6}, P_{8}$ |
|  | **nMIR** | **D** | VLDSGTIAPKFNERYQHWMC | $P_{1}, P_{10}, P_{9}, P_{3}, P_{2}, P_{4}, P_{7}, P_{8}, P_{5}, P_{6}$ |
|  |  | **E** | VLDGSTIAPKFNERYQHWMC | $P_{10}, P_{1},P_{9}, P_{3}, P_{2}, P_{4}, P_{7}, P_{8}, P_{5}, P_{6}$ |
|  | **TE** | **DI** | CWMHRYQNFEATKVISGDLP | $P_{1}, P_{4},P_{8}, P_{10}, P_{3}, P_{9},P_{7},P_{5}, P_{6}, P_{2}$ |
|  |  | **DO** | CMWHYIQNFRKADPSTELVG | $P_{3},P_{5}, P_{6},P_{9}, P_{7}, P_{2},P_{1},P_{10}, P_{4}, P_{8}$ |
|  |  | **K** | WCMHRYQANETFKVGISDLP | $P_{4},P_{3}, P_{8}, P_{1},P_{10}, P_{7}, P_{9},P_{5}, P_{6},P_{2}$ |
|  |  | **P** | WCMHRYQANETFKVGISDLP | $P_{4},P_{3}, P_{8}, P_{1},P_{10}, P_{7}, P_{9},P_{5}, P_{6},P_{2}$ |
| **Mi10ed** $\boldsymbol{\alpha}$ **and** $\boldsymbol{\beta}$ | **CR** | **D** | LEADVITPRGFSKNYQMHCW | $P_{2}, P_{10}, P_{5}, P_{4}, P_{1}, P_{3}, P_{9}, P_{8}, P_{7}, P_{6}$ |
|  |  | **E** | LEAVDITPRGFSKNYQMHCW | $P_{2}, P_{5}, P_{4}, P_{10}, P_{1}, P_{3}, P_{9}, P_{8}, P_{7}, P_{6}$ |
|  | **nMIR** | **D** | GLVEADISTPRFKHNQYWMC | $P_{2}, P_{5}, P_{10}, P_{1}, P_{9}, P_{4}, P_{3}, P_{7}, P_{6}, P_{8}$ |
|  |  | **E** | GLVEADISTPRFKHNQYWMC | $P_{2}, P_{5}, P_{10}, P_{1}, P_{9}, P_{4}, P_{3}, P_{7}, P_{6}, P_{8}$ |
|  | **TE** | **DI** | WMCHYQNFTRKVIPLDSAEG | $P_{3}, P_{2},P_{8}, P_{9}, P_{10}, P_{1}, P_{4}, P_{5}, P_{7}, P_{6}$ |
|  |  | **DO** | WMCHQYNPFTSKGVREDAIL | $P_{10}, P_{4},P_{7},P_{6},P_{9}, P_{1}, P_{3}, P_{2}, P_{5}, P_{8}$ |
|  |  | **K** | WMCYHQNVIKFRTLPDSAEG | $P_{3},P_{2},P_{8}, P_{10}, P_{4},P_{5}, P_{9},P_{1}, P_{7}, P_{6}$ |
|  |  | **P** | WMCYHQNVIFKRTLPDSAEG | $P_{3},P_{2},P_{8}, P_{10}, P_{4},P_{5}, P_{9},P_{1}, P_{7}, P_{6}$ |

**Table B. Centrality orders for the networks of D and APF features (CATH).** This table shows the centrality orders for the networks of D and APF features (CATH dataset) detected by pairwise Welch T-tests with significance level $\theta=0.05$. In the list of features (represented by abbreviations of amino acids) the left most features admit the significant highest ($P<0.05$) centralities among all features, while the right most features admit the significant lowest centralities among all features. The centralities are listed in descending orders (from left to right) according to the pairwise Welch T-tests. The full names and abbreviations of the 20 types of amino acids are presented in Supporting Information S1. The centrality orders for the other $\theta$ values can be found in Supporting Information S5 Dataset.

| **Structural**  **classes** | **Measures** | | **Centrality orders for networks of PseAAC features (CATH)** | |
| --- | --- | --- | --- | --- |
|  |  |  | **PseAAC (**$\boldsymbol{\lambda=0}$**)** | **PseAAC (**$\boldsymbol{\lambda=10}$**)** |
| **Mainly** $\boldsymbol{\alpha}$ | **CR** | **D** | KEGAPLRNIYVQCFWSDTHM | 12653981047RNEQLSKTGAPVICFWYHMD |
|  |  | **E** | KEGAPNRLIYVCQWFSTDHM | 12653910847RNSQTLVEIAGPKCFWHMYD |
|  | **nMIR** | **D** | EKNRLVDASIGFTYHMWPCQ | R2651103794E8VKSTDIFNAHGLYMWCQP |
|  |  | **E** | EKNRLVDASGITFYHMWPCQ | 265R11039478EVSKTNDIFHAGLYMWCQP |
|  | **TE** | **DI** | MWHFDCTLRVGIYSENKAPQ | MCWHPYQGFT83A4172K10D6L9I5VNSER |
|  |  | **DO** | MWCHIYSATLDGPKEFVRQN | MCWHPQG1310482975YNFL6IATSDKEVR |
|  |  | **K** | MWHFDTCLRVIGYESNAKPQ | MCWPQHG13Y8A5L210976F4TDKINVSER |
|  |  | **P** | MWHFDTCLRVGIYESNAKPQ | MCWPHQG1Y38A52L109F764TDKINVSER |
| **Mainly** $\boldsymbol{\beta}$ | **CR** | **D** | KENTLIRGSAVYPCDWHQFM | 25678910314TRSNAGVEIKWPLCQHYDFM |
|  |  | **E** | KENTRLISAGPYVWDCHQFM | 25867931014TRSNAVGIQWEPCLKHYDFM |
|  | **nMIR** | **D** | EGKSNTLFRAVPIQWYDHMC | 62758910431RESTNGVKLPFQAIYWDMHC |
|  |  | **E** | EKGSTNLFRAPVIQWYDHMC | 25678910431RTSENVGLPQKFAIWYDHMC |
|  | **TE** | **DI** | MWPHCGNYFDQKRSLATEIV | MCWHNDFQYLP3IGKRT5101EA6SV78924 |
|  |  | **DO** | MWCIDHRALQVKYNTSPEFG | MCWH53AT7182109ID6YSRL4QVGKPNFE |
|  |  | **K** | MWPHGFDNCYQKRLTSAEIV | MCWH35DNT1Y10P7LQAF8IGR6KS24VE9 |
|  |  | **P** | MWPHGFCNDYQKRLSTAEIV | MCWH3DN5T1YPQ10LFA78IGR6KSVE249 |
| **Mi10ed** $\boldsymbol{\alpha}$ **and** $\boldsymbol{\beta}$ | **CR** | **D** | KANIREGVLPYFSQWHCTDM | 62519310874RNASTKIQELPVGWCYHFDM |
|  |  | **E** | KANIRGPYELVFSQWHCTDM | 62511093874RNSTQIAKLEPVCWGHYDMF |
|  | **nMIR** | **D** | KAINREVLPSYGFQWDHTCM | R92651103874KNISETAQVPLYFWGDHCM |
|  |  | **E** | AKRINEPLVYGSFQWDHTMC | R96251103874NKSTIQEAVLPYFWGDHCM |
|  | **TE** | **DI** | MWNRFQKVYEDLPSIGATHC | MCWHYNQA4KD3RL8VGSF972T16105EPI |
|  |  | **DO** | MWHGQTSFADCNPYLERVKI | MWCGH810421576T39QDALENSYIPKFVR |
|  |  | **K** | MWNRQVKFPYELDGISAHTC | MCWH438N679RY2G105DQ1TKALSFPVEI |
|  |  | **P** | MWNRQFVKYPELDGISAHTC | MCWH43N8YR6792G105QDK1ATLSFPVEI |

**Table C. Centrality orders for the networks of PseAAC features (CATH).** This table shows the centrality orders for the networks of PseAAC features (CATH dataset) detected by pairwise Welch T-tests with significance level $\theta=0.05$. In the list of features (represented by abbreviations of amino acids and the integer tier lengths of the PseAAC features) the left most features admit the significant highest ($P<0.05$) centralities among all features, while the right most features admit the significant lowest centralities among all features. The centralities are listed in descending orders (from left to right) according to the pairwise Welch T-tests. The full names and abbreviations of the 20 types of amino acids are presented in Supporting Information S1. The centrality orders for the other $\theta$ values can be found in Supporting Information S5 Dataset.
