# Supplementary figures and images for: A protein structural study based on the centrality analysis of protein sequence feature networks
Source: PLoS One. 2021 Mar 29;16(3):e0248861. doi: 10.1371/journal.pone.0248861 (PMC8006989; doi:10.1371/journal.pone.0248861)

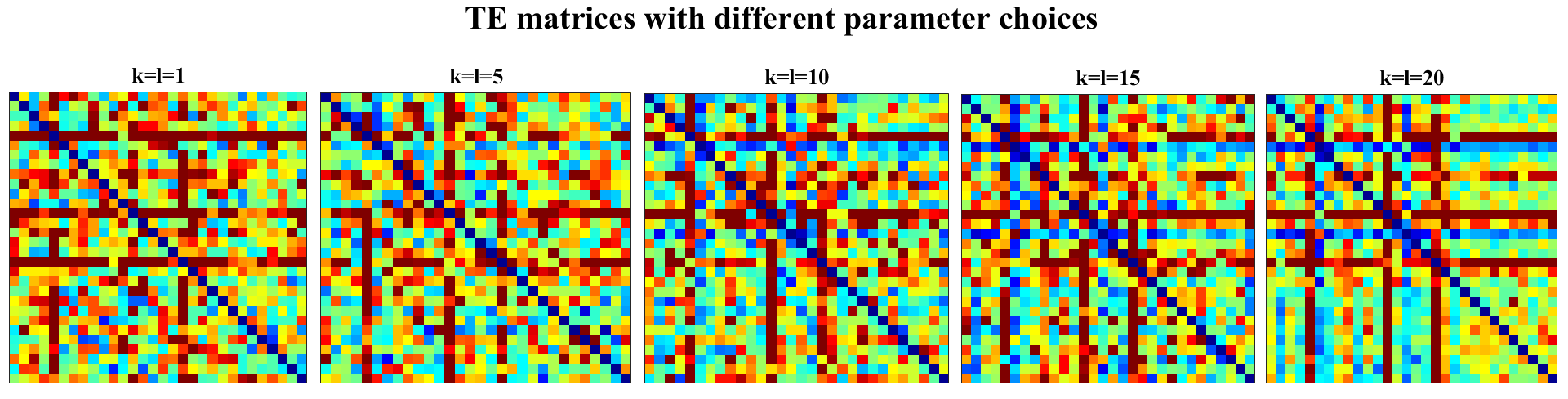

Supplement: S1 Fig — In this figure, the color matrices present the TE results computed with different embedding parameters. We can see that the different parameters of TE present similar results. (TIF) [file pone.0248861.s007.tif]
